# Tiger beetles (Coleoptera, Cicindelidae) of Northern Mindanao region (Philippines): checklist, distributional maps, and habitats

**DOI:** 10.3897/zookeys.1017.34500

**Published:** 2021-02-12

**Authors:** Dale Ann P. Acal, Jürgen Wiesner, Olga M. Nuñeza, Radomir Jaskuła

**Affiliations:** 1 Department of Biological Sciences, College of Science and Mathematics, Mindanao State University-Iligan Institute of Technology, Andres Bonifacio Ave., Tibanga, Iligan City 9200, Philippines Mindanao State University-Iligan Institute of Technology Iligan City Philippines; 2 Dresdener Ring 11, D-38444, Wolfsburg, Germany Unaffiliated Wolfsburg Germany; 3 Department of Invertebrate Zoology and Hydrobiology, Faculty of Biology and Environmental Protection, University of Lodz, Banacha 12/16, 90-237, Łódź, Poland University of Lodz Łódź Poland

**Keywords:** Endemic species, *
Calomera
*, *
Cylindera
*, diversity, distribution, *
Heptodonta
*, identification key, *
Lophyra
*, *
Neocollyris
*, *
Protocollyris
*, *
Prothyma
*, *
Therates
*, *
Tricondyla
*

## Abstract

The knowledge about tiger beetle fauna of the Northern Mindanao region (Philippines) is summarized based on literature data and new records. Thirty species classified in ten genera (*Tricondyla*, *Neocollyris*, *Protocollyris*, *Therates*, *Prothyma*, *Heptodonta*, *Thopeutica*, *Lophyra*, *Calomera*, and *Cylindera*) were documented from the area (56% of tiger beetle fauna of Mindanao and 21% of Philippine species). Twelve species were noted from Northern Mindanao region for the first time, including five taxa, *Neocollyris speciosa*, *Calomera angulata*, *Cylindera minuta*, *Lophyra striolata tenuiscripta*, and *Thopeutica virginea*, not recorded from Mindanao before. Distribution maps for all recorded species and the first photographs of habitats for some species in Mindanao and/or in the Philippines are provided. Eight species (27% of recorded fauna) were noted from riverine habitats while 18 tiger beetles (60%) were typical forest taxa; in the case of four species, their habitats in Northern Mindanao region are not known.

## Introduction

Tiger beetles (Coleoptera: Cicindelidae) are a beetle family (López-López and Vogler 2017; Duran and Gough 2020) of more than 2850 species distributed world-wide, but with the larger number of taxa occurring in tropical regions (Cassola and Pearson 2000; Wiesner 2020). With 162 taxa (including 144 species) actually known from the country the tiger beetle fauna of the Philippines is recognized as one of the most diverse in the world (Cabras et al. 2016a; Dheurle 2016, 2019; Zettel and Pangantihon 2017; Zettel and Wiesner 2018; Anichtchenko and Medina 2019, 2020; Medina et al. 2019, 2020c; Görn 2020). Moreover, it can be characterized by high percentage of endemic species as more than 85% of Cicindelidae are noted only from this country (Cassola and Pearson 2000; Cabras et al. 2016a; Dheurle 2016, 2019; Zettel and Pangantihon 2017; Zettel and Wiesner 2018; Anichtchenko and Medina 2019, 2020; Medina et al. 2019, 2020c; Görn 2020), with particular species often noted only on single islands (Cabras et al. 2016a). It can be expected that such high diversity values results from both geographical location of the country in the tropical region as well as the occurrence of a large number of geographically isolated islands that influence the evolution of endemic species.

Based on previous studies, ten genera and 54 species of tiger beetles were recorded on Mindanao Island: *Tricondyla* Latreille, 1822 (7 species), *Protocollyris* Mandl, 1975 (3 species), *Neocollyris* Horn, 1901 (12 species), *Therates* Latreille, 1817 (4 species), *Prothyma* Hope, 1838 (6 species), *Heptodonta* Hope, 1838 (2 species), *Calomera* Motschulsky, 1862 (3 species), *Lophyra* Motschulsky, 1859 (1 species), *Thopeutica* Chaudoir, 1861 (8 species), and *Cylindera* Westwood, 1831 (4 species) (Wiesner 1992; Cassola 2000, 2011; Naviaux 1994, 2002; Cassola and Ward 2004; Dheurle 2015, 2019; Cabras et al. 2016a; Anichtchenko and Medina 2019; Görn 2020; Medina et al. 2019, 2020c).

Although more than 50 tiger beetle species are known as occurring on Mindanao Island (36% of Philippine fauna), for many of these taxa only single records are known (Cabras et al. 2016a). Hence, little is known about the general distribution of many species and from many regions no data are available. As Philippine tiger beetle fauna includes many endemic taxa, lack of distributional data often do not allow estimates of species ranges, abundance, or habitat selection and as a consequence, also their threats. As degradation of wildlife both in the Philippines and Mindanao is significant due to different human activities (e.g., deforestation for agricultural land, human pressures because of overpopulation; Lasco et al. 2008; Navarrete et al. 2018), even simple faunistic data may play an important role in the conservation of this beetle group.

In the present paper we focus on the region of Northern Mindanao where no regular studies on tiger beetle species were previously done. As a result, the first checklist of Cicindelidae occurring in this region as well as distributional maps for all known species occurring in the area, and photographs of habitats for the 12 recorded taxa are provided. This paper may serve as a baseline for further studies on this beetle family not only in the Northern Mindanao but also in other regions in the country.

## Materials and methods

**Figure 1.**
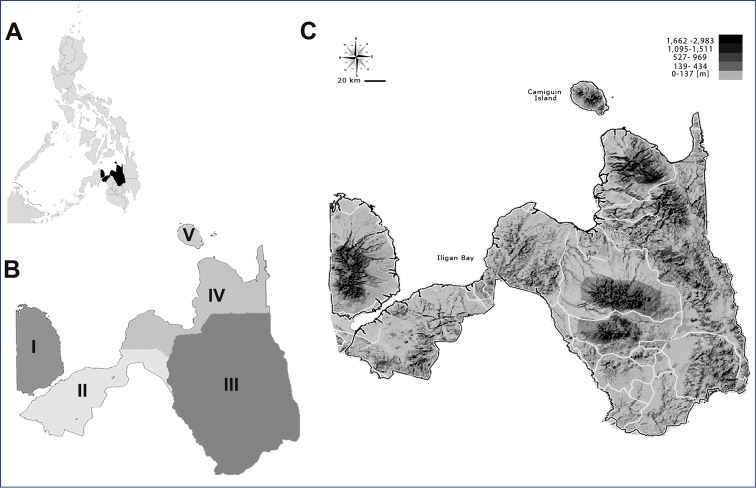
Map of Northern Mindanao region **A** in relation to entire Mindanao island and the remaining Philippine archipelago **B** administrative provinces (I – Misamis Occidental, II – Lanao del Norte, III – Bukidnon, IV – Misamis Oriental, V – Camiguin) **C** detailed physical map.

**Figure 2.**
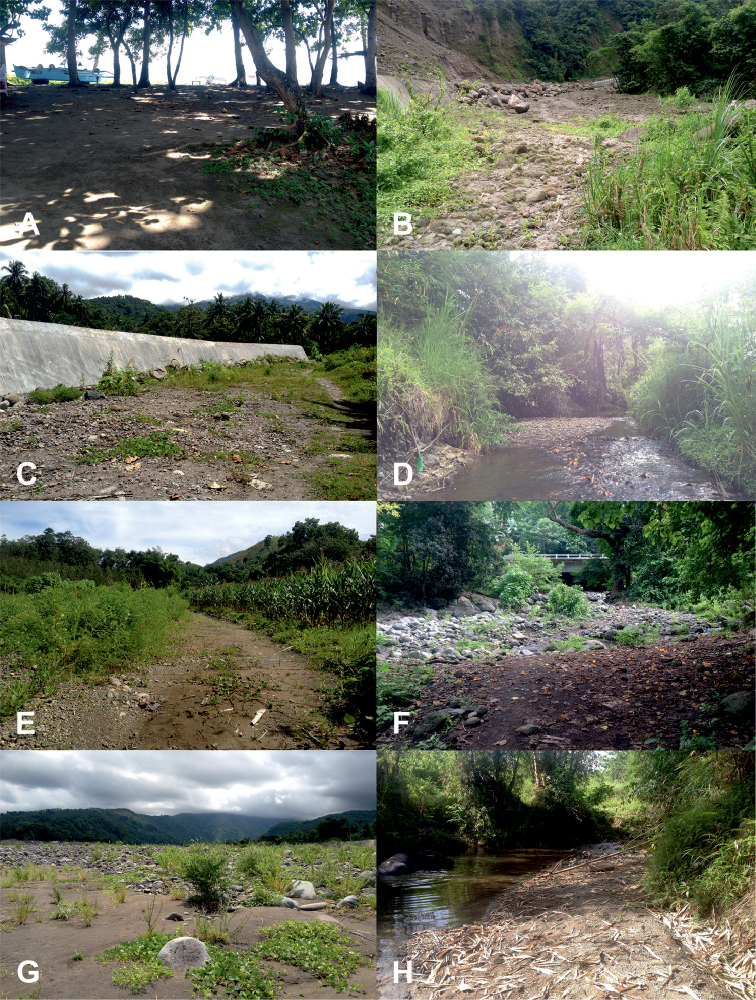
Habitats of tiger beetles from Northern Mindanao region: *Calomera mindanaoensis* (**A–G**) (sites 1–7), *Cylindera discreta elaphroides* (**H**) (site 8), *C. minuta* (**G**) (site 7), *C. mouthiezi* (**H**) (site 8) (descriptions of sites in Table 1; photographs DAPA).

**Figure 3.**
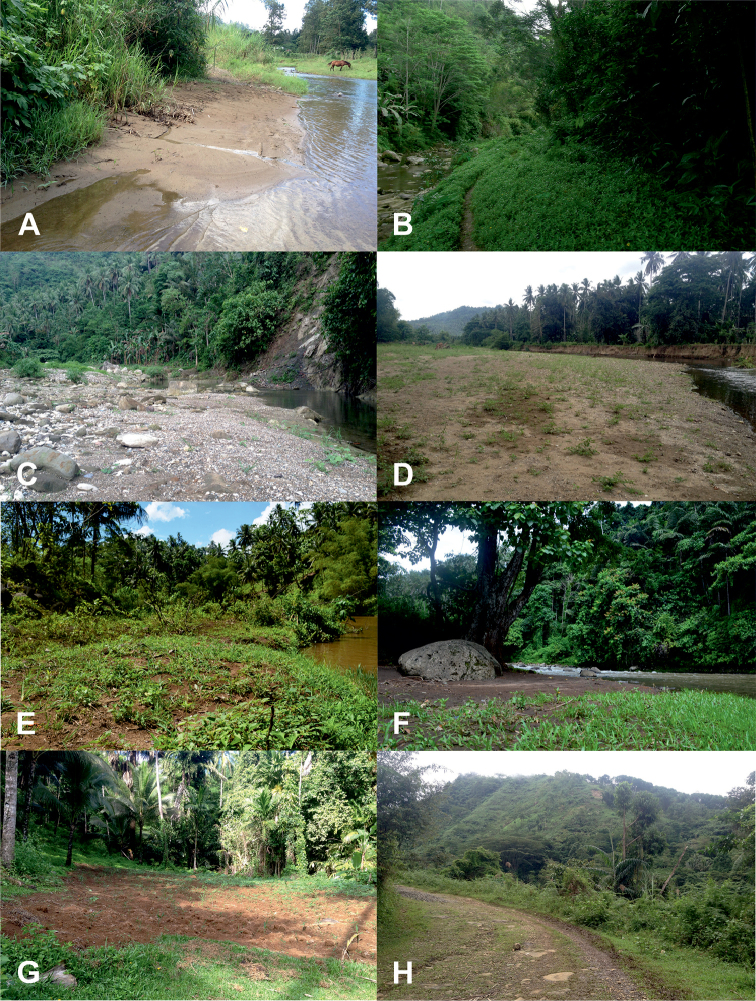
Habitats of tiger beetles from Northern Mindanao region: *Calomera angulata angulata* (**C–E**) (sites 10–12), *C. lacrymosa* (**C, E**) (sites 10, 12), *C. mindanaoensis* (**C–F, H**) (sites 10–13, 15), *Cylindera discreta elaphroides* (**A, C–E**) (sites 9–12), *C. minuta* (**A, C–E**) (sites 9–12), *C. viduata* (**G**) (site 14), *Lophyra striolata tenuiscripta* (**G**) (site 14), *Therates coracinus coracinus* (**B**) (site 10) (descriptions of sites in Table 1; photographs **A, C–H** DAPA, **B** RJ).

**Figure 4.**
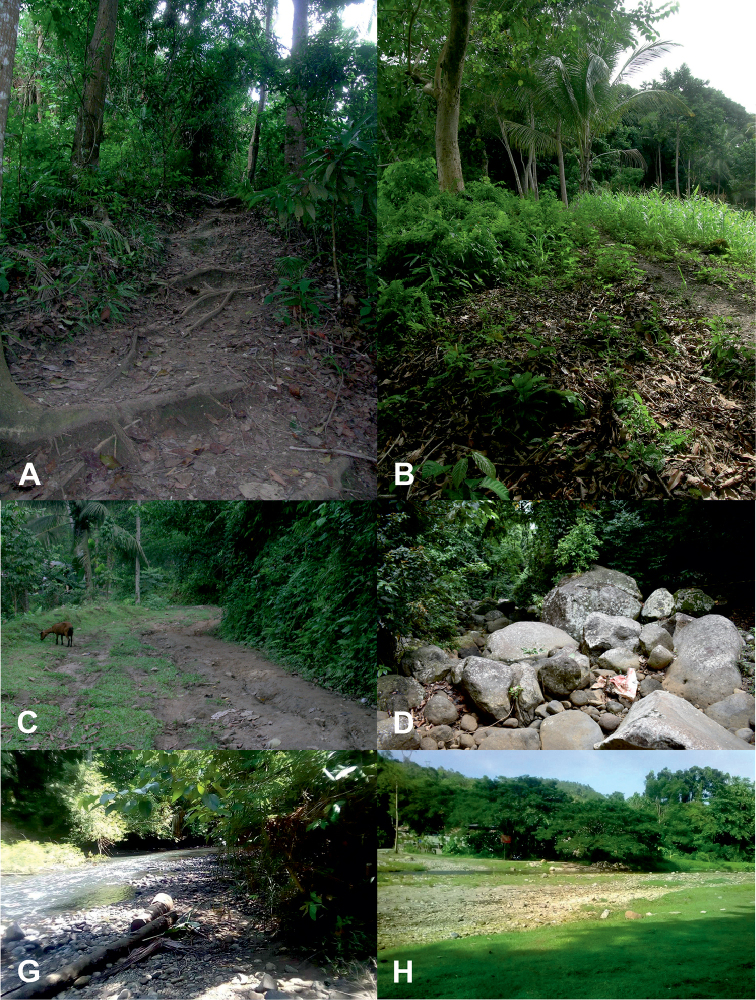
Habitats of tiger beetles from Northern Mindanao region: *Calomera mindanaoensis* (**C**) (site 16) (**E, F**) (sites 18–19), *Prothyma heteromallicollis heteromallicollis* (**A**) (site 16), *Neocollyris similior* (**D**) (site 17), *Therates coracinus coracinus* (**D**) (site 17), *T. fasciatus fasciatus* (**D**) (site 17), and *Tricondyla aptera punctipennis* (**B**) (site 16) (descriptions of sites in Table 1; photographs **A–C** RJ, **D** O. Bagona, **E** A.B. Lapore, **F** C. Torres).

Northern Mindanao geographically lies within latitude 7°15' to 9°15'N and longitude 123°30' to 125°30'E, is bound on the north by the Bohol Sea, on the west by Zamboanga provinces, on the east by Agusan and Davao provinces, on the south by Lanao del Sur and Cotabato. The whole region covers a total land area of 19,279.60 km² and more than 60% of the region’s area are classified as forest land. Geologically, this region is formed of a combination of coastal areas, rivers, falls, volcanoes, highlands with flat terrain, rugged and faulted mountains with rich soil, abundant minerals, and agricultural resources. Since the region is located outside the typhoon belt area, rainfall is evenly distributed throughout the year (Dejarme-Calalang and Colinet 2014; Bouquet 2017). Northern Mindanao comprises five provinces: Bukidnon, Camiguin, Lanao del Norte, Misamis Occidental, and Misamis Oriental (Figure 1). Regular studies were done in sixteen sampling sites in the Northern Mindanao region, including riverine areas, secondary forests, and coastal areas (Table 1, Figures 2–4), and most of the tiger beetle material used in this study was collected using entomological hand nets during field work in 2017–2019. Some additional data were provided from earlier studies, including published data (Wiesner 1988; Naviaux 1994, 2002; Cassola 2000, 2011; Dheurle 2015, 2017; Cabras et al. 2016a; Görn 2020).

**Table 1.** Sampling sites in Northern Mindanao region were regular studies were done in years 2017 and/or 2018.

Site	Sampling site	GPS coordinates
1.	Barangay Bulokbulok, Municipality of Mambajao, Camiguin Island, Camiguin Province	9°15'9"N, 124°42'31"E
2.	Looc River, Barangay Mainit, Municipality of Catarman, Camiguin Island, Camiguin Province	9°10'30"N, 124°40'44"E
3.	Sagay River, Barangay Bonbon, Municipality of Sagay, Camiguin Island, Camiguin Province	9°6'18"N, 124°43'57"E
4.	Barangay Bura, Municipality of Catarman, Camiguin Island, Camiguin Province	9°10'4.7"N, 124°39'23"E
5.	Barangay Poblacion, Municipality of Mambajao, Camiguin Island, Camiguin Province	9°13'24"N, 124°41'47"E
6.	Barangay Umagus, Municipality of Lagonglong, Misamis Oriental Province	8°48'11"N, 124°48'53"E
7.	Cabulaway River, Municipality of Balingasag, Misamis Oriental Province	8°46'9"N, 124°48'2"E
8.	Barangay Kalasungay, Malaybalay City, Bukidnon Province	8°11'28"N, 125°5'54"E
9.	Barangay Can-ayan, Malaybalay City, Bukidnon Province	8°11'31"N, 125°9'13"E
10.	Barangay Bonbonon, Iligan City, Lanao del Norte Province	8°15'56"N, 124°18'37"E
11.	Tubod River, Barangay Merilla, Iligan City, Lanao del Norte Province	8°12'17"N, 124°15'24"E
12.	Barangay Esperanza, Municipality of Bacolod, Lanao del Norte Province	8°10'12"N, 124°0'22"E
13.	Barangay Mati, Municipality of Bacolod, Lanao del Norte Province	8°9'4"N, 124°0'57"E
14.	Barangay San Isidro, Municipality of Sinacaban, Misamis Occidental Province	8°17'5"N, 123°47'5"E
15.	Barangay San Lorenzo, Municipality of Sinacaban, Misamis Occidental Province	8°17'10"N, 123°41'43"E
16.	Mt. Agad-Agad, Iligan City, Lanao del Norte Province	8°12'49.34"N, 124°16'9.66"E
17.	Mimbilisan Protected Landscape, Misamis Oriental Province	8.94884N, 124.86517E
18.	Municipality of Lopez Jaena, Misamis Occidental Province	8°33'00"N, 123°46'00"E

Material is currently deposited in the authors’ collections:

**DAC** D.A.P. Acal Collection (Illigan City, Philippines);

**JWC** J. Wiesner Collection (Wolfsburg, Germany);

**RJC** R. Jaskuła Collection (Łódź, Poland).

## Checklist of tiger beetles of Northern Mindanao

### Family Cicindelidae Latreille, 1802

**Subfamily Cicindelinae Latreille, 1802**

**Tribus Collyridini Brullé, 1834**

**Subtribus Tricondylina Naviaux, 1991**

#### Genus *Tricondyla* Latreille, 1822

The genus *Tricondyla* is represented in the Philippine islands by 14 species, including seven recorded from Mindanao Island (Naviaux 2002; Cabras et al. 2016a).

#### Subgenus Tricondyla Latreille, 1822

##### Tricondyla (Tricondyla) apterapunctipennis

Taxon classificationAnimaliaColeopteraCicindelidae

Chevrolat, 1841

32D97F20-5202-58FC-A067-0026CDE0E879

**Figure 5.**
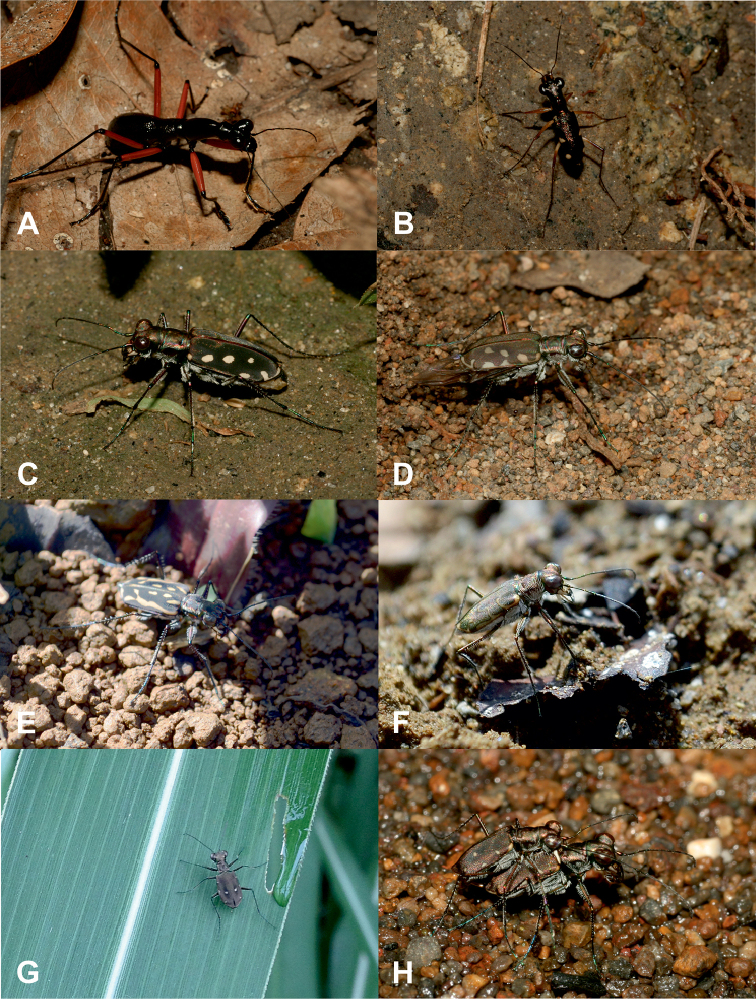
Some of the tiger beetle species recorded in Northern Mindanao region: **A**Tricondyla (Tricondyla) aptera punctipennis**B**Prothyma (Symplecthyma) heteromallicollis heteromallicollis**C***Calomera mindanaoensis***D***C. lacrymosa***E**Lophyra (Spilodia) striolata tenuiscripta**F**Cylindera (Eugrapha) minuta**G, H**C. (Ifasina) discreta elaphroides (photographs **A–D, H** RJ, **E–G** DAPA).

**Figure 6.**
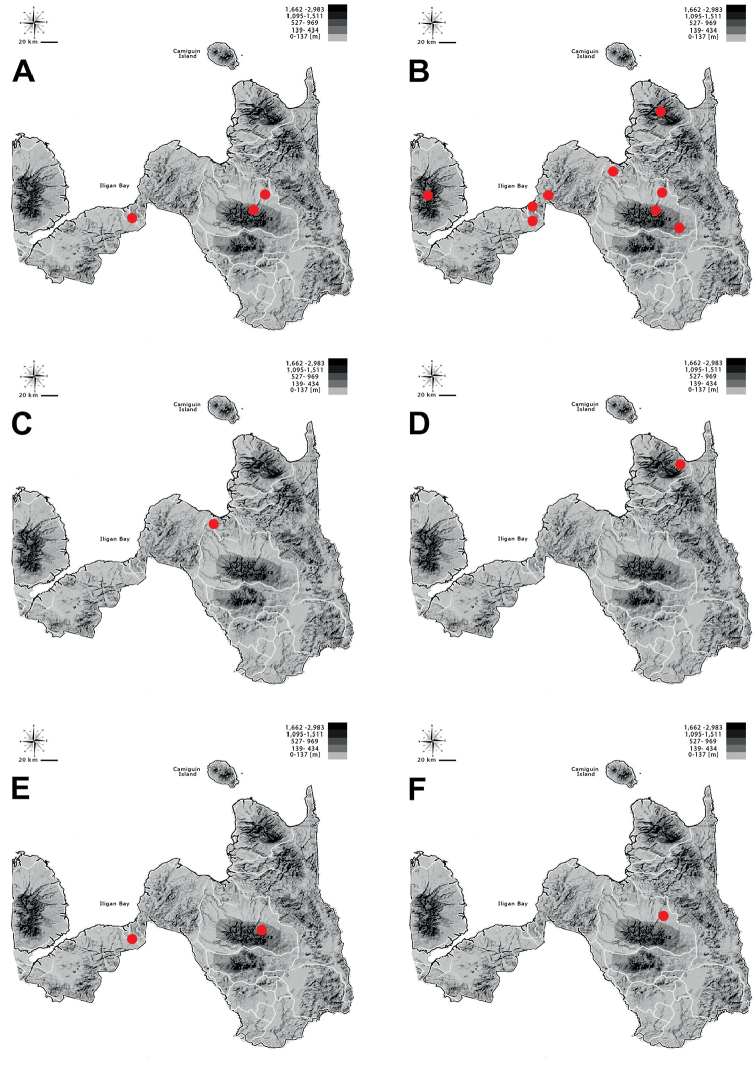
Distribution of **A**Tricondyla (Tricondyla) aptera punctipennis**B**T. (T.) elongata**C**T. (T.) gracilis**D**T. (Stenotricondyla) cyanipes**E**T. (Stenotricondyla) cavifrons, and **F**Neocollyris (Neocollyris) albitarsis in Northern Mindanao region.

Figures 4B , 5A , 6A

###### General distribution.

Subspecies known from Indonesia and Philippines. In the Philippines it was found in Sibuyan, Samar, Cebu, and Mindanao; in Mindanao Island it was recorded only in the Northern Mindanao region (Cabras et al. 2016a).

###### Literature data for Northern Mindanao.

**Bukidnon province**: Impasung-ong (Cabras et al. 2016a).

###### Material examined.

**Bukidnon Province**: Mt. Kitanglad, 28.VII.1990, 1♂ 1♀, ex coll. Y. Nishiyama (JWC); [no detailed locality], 1977, 1♂, leg. R. Lumawig (JWC); **Lanao del Norte province**: Mt. Agad-agad, 8°12'49.34"N, 124°16'9.66"E, 470 m a.s.l., 19.11.2018, 1♂, leg. R. Jaskuła et D.A.P. Acal (RJC).

###### Habitat.

Forest species found on vertical and fallen tree trunks, sometimes in forest floor; occasionally also outside the forest (but close to the trees).

###### Remarks.

Larva of nominal subspecies was described by Trautner and Schawaller (1996), who observed it hunting during night period in the bark of tree (e.g., *Samanea saman*).

##### Tricondyla (Tricondyla) elongata

Taxon classificationAnimaliaColeopteraCicindelidae

Horn, 1906

2CFBD358-0E05-51A1-B900-34F92A9D71D8

Figure 6B

###### General distribution.

Species endemic to the Philippines, where it was recorded from Luzon, Visayas, and Mindanao; in Mindanao recorded only in Northern Mindanao, Davao, and Bangsamoro Autonomous Region in Muslim Mindanao regions (Cabras et al. 2016a, b; Marohomsalic et al. 2021).

###### Literature data for Northern Mindanao.

**Bukidnon province**: Lantapan and Impasung-ong (Cabras et al. 2016a); **Misamis Occidental province**: Mt. Malindang (Cabras et al. 2016a); **Misamis Oriental province**: Mt. Balatucan-Lumot (Cabras et al. 2016a).

###### Material examined.

**Bukidnon province**: Mt. Kitanglad, 28.VII.1990, 1♂ 2♀♀, ex coll. Y. Nishiyama (JWC); Mt. Kitanglad, 10.2014, 9♂♂ 5♀♀, leg. N. Mohagan (JWC); **Lanao del Norte province**: Mount Agad-agad, 8°12'49.34"N, 124°16'9.66"E, ca. 470 m a.s.l., 11.2019, 1 ex., leg. J. Ebina, M. L. Lumontod, G. C. Café (RJC); Dodiongan Falls, Iligan City – Barangay Bonbonon, 8.271457N, 124.314140E, 47 m a.s.l., 11.2019, 1 ex., leg. R. Jaskuła (RJC); Tinago Falls, Iligan City – Barangay Ditucalan, 8.159820N, 124.185460E, 11.2019, 1 ex., leg. Ł. Trębicki (RJC); **Misamis Oriental province**: Cagayan de Oro, Malasag forest, 23.05.1978, 1♀, leg. A. Bandinelli (JWC).

###### Habitat.

Forest, arboreal species found on vertical and fallen tree trunks. Marohomsalic et al. (2021) recorded native and invasive tree species having extrafloral nectaries as favorite hunting areas for this species in the human-disturbed habitats.

##### Tricondyla (Tricondyla) gracilis

Taxon classificationAnimaliaColeopteraCicindelidae

Naviaux, 2002

AD2FDDC8-A470-57C0-8AFD-664BC8B8E579

Figure 6C

###### General distribution.

Species endemic to Philippines where it was found only in Mindanao and Romblon islands (Naviaux 2002; Cabras et al. 2016a); in Mindanao known from Davao and Northern Mindanao regions (Naviaux 2002).

###### Literature data for Northern Mindanao.

**Misamis Oriental province**: Malasag forest Cagayan de Oro (Naviaux 2002).

###### Habitat.

Forest, arboreal species found on vertical and fallen tree trunks.

#### Subgenus Stenotricondyla Naviaux, 2002

##### Tricondyla (Stenotricondyla) cyanipes

Taxon classificationAnimaliaColeopteraCicindelidae

Eschscholtz, 1829

096A64BA-B7A3-5DA6-BC42-BD553550C082

Figure 6D

###### General distribution.

Species endemic to the Philippines where it was found in Luzon, Leyte, Sibuyan, and Mindanao; in Mindanao recorded only in Northern Mindanao region (Cabras et al. 2016a).

###### Literature data for Northern Mindanao.

**Misamis Oriental province**: Gingoog City (Cabras et al. 2016a).

###### Habitat.

Forest, arboreal species found on vertical and fallen tree trunks.

##### Tricondyla (Stenotricondyla) cavifrons

Taxon classificationAnimaliaColeopteraCicindelidae

Schaum, 1862

E68C4B65-1F52-5EDB-A8FA-D33BC3C02727

Figure 6E

###### General distribution.

Species endemic to the Philippines where it was noted from Balabac, Mindanao, and Palawan; in Mindanao it was recorded only from Bangsamoro Autonomous Region in Muslim Mindanao (Marohomsalic et al. 2021), this is the first record from Northern Mindanao region.

###### Material examined.

**Bukidnon province**: Mt. Kitanglad, 10.2014, 1♀, leg. N. Mohagan (JWC); Mt. Kitanglad, 11–12.2014, 2♂♂ 1♀, leg. N. Mohagan (JWC); **Lanao del Norte province**: Mount Agad-agad, 8°12'49.34"N, 124°16'9.66"E, ca. 470 m a.s.l., 11.2019, 3 exx., leg. J. Ebina, M. L. Lumontod, G. C. Café (RJC).

###### Habitat.

Forest, arboreal species found on vertical and fallen tree trunks. Marohomsalic et al. (2021) recorded native and invasive tree species having extrafloral nectaries as favorite hunting areas for this species.

#### Genus *Neocollyris* Horn, 1901

The genus *Neocollyris* is represented in the Philippines by 29 species, including 12 recorded from Mindanao Island (Naviaux 1994; Cabras et al. 2016a).

#### Subgenus Neocollyris Horn, 1901

##### Neocollyris (Neocollyris) albitarsis

Taxon classificationAnimaliaColeopteraCicindelidae

(Erichson, 1834)

FDC4EBD8-ED85-5B87-9EAF-2F4DB8604487

Figure 6F

###### General distribution.

Species endemic to the Philippines, noted from Homonhon, Luzon, Mindanao, and Palawan; in Mindanao it was recorded only from Northern Mindanao and Bangsamoro Autonomous Region in Muslim Mindanao region (Cabras et al. 2016a; Marohomsalic et al. 2021).

###### Literature data for Northern Mindanao.

**Bukidnon province**: Impasung-ong (Cabras et al. 2016a).

###### Habitat.

Forest, arboreal species found on vertical and fallen tree trunks and leaves of different bush species.

##### Neocollyris (Neocollyris) brevicula

Taxon classificationAnimaliaColeopteraCicindelidae

Naviaux, 1994

9210F89A-FF31-52F3-8512-4B95A77173D9

Figure 7A

###### General distribution.

Species endemic to Philippines, known from Basilan, Mindanao, and Samar island (Cabras et al. 2016a); in Mindanao recorded only in Northern Mindanao (Naviaux 1994).

###### Literature data for Northern Mindanao.

**Lanao del Norte province**: Municipality of Balo-i, Barangay Momungan (Naviaux 1994).

###### Habitat.

Forest, arboreal species found on vertical and fallen tree trunks and leaves of different bush species.

##### Neocollyris (Neocollyris) emarginata

Taxon classificationAnimaliaColeopteraCicindelidae

(Dejean, 1825)

3A98B29A-66B9-584E-8453-8F165E43767E

Figure 7B

###### General distribution.

Species noted from Borneo, Indonesia, Malaysia, and Philippines, where it was found in Mindanao and Palawan; in Mindanao recorded only in Northern Mindanao region (Cabras et al. 2016a).

###### Literature data for Northern Mindanao.

**Bukidnon province**: Impasug-ong (Cabras et al. 2016a).

###### Habitat.

Forest, arboreal species found on vertical and fallen tree trunks and leaves of different bush species.

#### Subgenus Heterocollyris Naviaux, 1995

##### Neocollyris (Heterocollyris) affinis

Taxon classificationAnimaliaColeopteraCicindelidae

(Horn, 1892)

00BF7A1F-8061-50ED-96C8-9B351D055CFA

Figure 7C

###### General distribution.

Species endemic to the Philippines where it was recorded in Bohol, Leyte, Luzon, Mindanao, Panay, and Samar; in Mindanao found only in Northern Mindanao region (Cabras et al. 2016a).

###### Literature data for Northern Mindanao.

**Bukidnon province**: Impasug-ong (Cabras et al. 2016a); **Misamis Oriental province**: Balatucan-Lumot (Cabras et al. 2016a).

###### Habitat.

Forest, arboreal species found on vertical and fallen tree trunks and leaves of different bush species.

##### Neocollyris (Heterocollyris) similior

Taxon classificationAnimaliaColeopteraCicindelidae

(Horn, 1893)

C452B52D-D9D6-53D6-AD39-47AB0BE9AE19

Figures 4D , 7D

###### General distribution.

Species endemic to Philippines where it was recorded only from Mindanao (Naviaux 1994; Cabras et al. 2016; Marohomsalic et al. 2021).

###### Material examined.

**Misamis Oriental province**: Mimbilisan Protected Landscape, 8.94884N, 124.86517E, 501 m a.s.l., 18.07.2017, 1♂, leg. O. Bagona (RJC); **Lanao del Norte province**: Dodiongan Falls, Iligan City – Barangay Bonbonon, 8.271457N, 124.314140E, 47 m a.s.l., 11.2019., 1 ex., leg. M. L. Lumontod (RJC).

###### Habitat.

Forest, arboreal species found on vertical and fallen tree trunks and leaves of different bush species.

##### Neocollyris (Heterocollyris) speciosa

Taxon classificationAnimaliaColeopteraCicindelidae

(Schaum, 1863)

C9DD9971-D3C5-504B-90C2-E4C9911AB151

Figure 7E

###### General distribution.

Species endemic to the Philippines, where it was noted only from Luzon, Mindoro (Cabras et al. 2016a), and Mindanao (new record).

###### Material examined.

**Bukidnon province**: Mt. Kitanglad, 11–12.2014, 2♀♀, leg. N. Mohagan (JWC).

###### Habitat.

Forest, arboreal species found on vertical and fallen tree trunks and leaves of different bush species.

#### Genus *Protocollyris* Mandl, 1975

##### Protocollyris mindanaoensis

Taxon classificationAnimaliaColeopteraCicindelidae

(Mandl, 1974)

32AFB5A7-5747-5117-B16F-C9F54E96DE48

Figure 7F

###### General distribution.

Species endemic for Philippines where it was noted only from Mindanao Island (Cabras et al. 2016a) from Northern Mindanao region (Naviaux 1994).

###### Literature data for Northern Mindanao.

**Lanao del Norte province**: Momungan [actually Barangay Momungan in Municipality of Balo-i] (Naviaux 1994).

###### Habitat.

Forest, arboreal species.

#### Tribe Cicindelini Latreille, 1802

**Subtribus Theratina Horn, 1910**

**Genus *Therates* Latreille, 1817** The genus *Therates* is represented in the Philippine islands by six species, including four or five recorded from Mindanao Island; three of them have been noted from the Northern Mindanao region by Cabras et al. (2016a).

##### Therates coracinus coracinus

Taxon classificationAnimaliaColeopteraCicindelidae

Erichson, 1834

9E71DEBD-0DE2-57F1-ADD8-798C7047898C

Figures 3B , 4D , 8A

###### General distribution.

Subspecies known from Indonesia, Moluccas, and Philippines, where it was recorded in Balabac, Leyte, Luzon, Mindanao, Mindoro, Negros, Palawan, Panay, Romblon, and Samar; in Mindanao recorded from Davao, Northern Mindanao, and Soccsksargen regions (Wiesner 1988; Cabras et al. 2016a, b; Cabras and Wiesner 2016; Pepito et al. 2020).

###### Literature data for Northern Mindanao.

**Bukidnon province**: Impasug-ong (Cabras et al. 2016a).

###### Material examined.

**Bukidnon province**: Mt. Kitanglad, 28.07.1990, 1♂ 1♀, ex coll. Y. Nishiyama (JWC); Mt. Kitanglad, 11–12.2014, 6♂♂ 6♀♀, leg. N. Mohagan (JWC); Mt. Kalatungan, Sitio Bato, Municipality of Maramag, 11.2019, 1 ex., leg. R. Jaskuła (RJC). **Lanao del Norte province**: Dodiongan Falls, Iligan City – Barangay Bonbonon, 8.271457N, 124.314140E, 47 m a.s.l., on Araceae leaves, 07.12.2018, 1♂, leg. R. Jaskuła (RJC), 11.2019, 6 exx., leg. D. A. P. Acal, J. Ebina, M. L. Lumontod, R. Jaskuła (RJC); Mount Agad-agad, 8°12'49.34"N, 124°16'9.66"E, ca. 470 m a.s.l., on leaves, 11.2019, 2 exx., D. A. P. Acal, J. Ebina (RJC). **Misamis Oriental province**: Mimbilisan Protected Landscape, 8.94884N, 124.86517E, 501 m a.s.l., 18.07.2017, 3 exx., leg. O. Bagona (RJC); Bolyok Falls, Barangay Lubilan, Naawan Municipality, 11.20218, 4 exx., leg. R. Jaskuła, D.A.P. Acal, J. Ebina, M. L. Lumontod (RJC); Mambuntan Falls, Barangay Lubilan, Naawan Municipality, 8.412300N, 124.351642E, 11.2019, 1 ex., leg. R. Jaskuła (RJC).

###### Habitat.

Forest species noted on tree trunks and leaves.

###### Remarks.

When disturbing, actively fast flying among trees; during flight shows bright orange abdomen coloration. Taxonomical status of both subspecies of *Therates coracinus* noted in Mindanao (spp. *coracinus* and ssp. fulvescens Wiesner, 1988) should be revised including molecular data as they probably represent separate species or synonyms.

#### *Therates fasciatus* (Fabricius, 1801)

##### Therates fasciatus fasciatus

Taxon classificationAnimaliaColeopteraCicindelidae

(Fabricius, 1801)

6ABFD659-654A-5D40-B9D4-4DB4426257DD

Figures 4D , 8B

###### General distribution.

Subspecies known from Indonesia and Philippines. In the Philippines recorded only from Mindanao and Palay islands (Cabras et al. 2016a); from Mindanao Island it was known only from Davao and Northern Mindanao regions (Cabras et al. 2016b).

###### Literature data for Northern Mindanao.

**Bukidnon province**: [no detailed locality] (Wiesner 1988).

###### Material examined.

**Bukidnon province**: [no detailed locality], 1977, 1♀, leg. R. Lumawig (JWC). **Camiguin province**: Camiguin Island, Municipality of Catarman, Mt. Timpoong-Hibok-Hibok Natural Monument, Mt. Hibok-Hibok 11.2019, 2 exx., leg. R. Jaskuła, D. A. P. Acal (RJC); **Lanao del Norte province**: Dodiongan Falls, Iligan City – Barangay Bonbonon, 8.271457N, 124.314140E, 47 m a.s.l., 11.2019, 11 exx., leg. R. Jaskuła, D.A.P. Acal, J. Ebina, M. L. Lumontod (RJC); near Sikyop Cave, Iligan City – Barangay Lawlawon, 8.246627N, 124.422387E, 11.2019, 2 exx., leg. R. Jaskuła (RJC); **Misamis Oriental province**: Mimbilisan Protected Landscape 8.94884N, 124.86517E, 501 m a.s.l., 18.07.2017, 13 exx., leg. O. Bagona (RJC); Bolyok Falls, Barangay Lubilan, Naawan Municipality, 11.2019, 1 ex., leg. R. Jaskuła (RJC).

###### Habitat.

Forest species noted on tree trunks and leaves.

##### Therates fasciatus pseudolatreillei

Taxon classificationAnimaliaColeopteraCicindelidae

Horn, 1928

DC647B67-C4C7-5EC6-9925-70A70AF0FD7F

Figure 8B

###### General distribution.

Subspecies endemic to Philippines where it was recorded from Leyte, Mindanao, and Mindoro; in Mindanao noted only from Northern Mindanao and Soccsksargen regions (Wiesner 1988; Cabras et al. 2016a; Pepito et al. 2020).

###### Literature data for Northern Mindanao.

**Bukidnon province**: Impasug-ong (Cabras et al. 2016a); **Lanao del Norte province**: “Mai-nit” (actually Barangay Mainit) (Wiesner 1988); **Misamis Occidental province**: Malindang Range (Cabras et al. 2016a).

###### Material examined.

**Bukidnon province**: Mt. Talemo, 30.06.1977, 1♂, leg. M. Sato (JWC); **Lanao del Norte province**: Barangay Mainit (between Iligan City and Cagayan de Oro), 17–20.08.1978, 2♂♂ 2♀♀, leg. Cabides et Lobin (JWC); **Misamis Oriental province**: Gingoog, 8.5N, 125.0E, 04.1984, 6♂♂ 4♀♀ (JWC).

###### Habitat.

Forest species noted on tree trunks and leaves.

###### Remarks.

Taxonomical status of all four subspecies of *Therates fasciatus* noted in Mindanao (spp. *fasciatus* (Fabricius, 1801), spp. *quadrimaculatus* Horn, 1895, spp. *pseudolatreillei* Horn, 1928, and ssp. flavohumeralis Mandl, 1964) should be revised including molecular data as probably at least some represent separate species or synonyms.

#### *Therates fulvipennis* Chaudoir, 1848

##### Therates fulvipennis bidentatus

Taxon classificationAnimaliaColeopteraCicindelidae

Chaudoir, 1861

03863137-6640-55CD-83AF-6F7F05F21CCA

Figure 8C

###### General distribution.

Subspecies known from Indonesia and Philippines (Basilan and Mindanao islands); from Mindanao noted from Northern Mindanao region only (Wiesner 1988; Cabras et al. 2016a).

###### Literature data for Northern Mindanao.

**Lanao del Norte province**: “Ma-Init” [actually Barangay Mainit] (Wiesner 1988).

###### Material examined.

**Bukidnon province**: Mt. Kitanglad, 10.2014, 15♂♂ 5♀♀, leg. N. Mohagan (JWC); **Lanao del Norte province**: Barangay Mainit (between Iligan City and Cagayan de Oro), 17–20.08.1978, 5♂♂ 3♀♀, leg. Cabides et Lobin (JWC).

###### Habitat.

Forest species noted on tree trunks and leaves.

##### Therates fulvipennis everetti

Taxon classificationAnimaliaColeopteraCicindelidae

Erichson, 1834

14CDB241-18F2-5EE4-A2B1-8CA1979685FF

Figure 8C

###### General distribution.

Subspecies endemic to the Philippines, where it was recorded from Dinagat, Luzon, Mindanao, Negros, and Panay; in Mindanao it was recorded in Davao, Northern Mindanao, and Soccsksargen regions (Cabras et al. 2016a, b).

###### Literature data for Northern Mindanao.

**Bukidnon province**: Impasug-ong (Cabras et al. 2016a).

###### Habitat.

Forest species noted on tree trunks and leaves.

###### Remarks.

Taxonomical status of all three subspecies of *Therates fulvipennis* noted in Mindanao (spp. *bidentatus* Chaudoir, 1861, ssp. fulvipennis Chaudoir, 1848, and spp. *everetti* Erichson, 1834) should be revised including molecular data as probably at least some of them represent separate species or synonyms.

#### Subtribus Dromicina Thomson, 1859

**Genus *Prothyma* Hope, 1838** The genus *Prothyma* is represented in the Philippine islands by 12 species, including six recorded from Mindanao Island (Cabras et al. 2016a).

##### Prothyma (Symplecthyma) heteromallicollisheteromallicollis

Taxon classificationAnimaliaColeopteraCicindelidae

Horn, 1909

9DC0D4D8-F135-592B-8369-7DFF06CB5CD6

Figures 4A , 5B , 8D

###### General distribution.

Species endemic to the Philippines, where it was recorded on Luzon and Mindanao till now (Cabras et al. 2016a); in Mindanao it has been noted from Davao (Cabras et al. 2016b) and Northern Mindanao (this publication) regions.

###### Material examined.

**Bukidnon province**: Mt. Kitanglad, 11–12.2014, 3♂♂ 1♀, leg. N. Mohagan (JWC); **Lanao del Norte province**: Mount Agad-agad, 8°12'49.34"N, 124°16'9.66"E, 470 m a.s.l., 18.10.2018, 10 exx., leg. D. A. P. Acal (DAC), 19.11.2018, 2♂♂, leg. R. Jaskuła et D. A. P. Acal (RJC); **Misamis Oriental province**: Mimbilisan Protected Area, 8.94884N, 124.86517E, 501 m a.s.l., 18.07.2017, 1♂, leg. O. Bagona (RJC).

###### Habitat.

Species found on shaded forest paths and forest litters.

###### Remarks.

Some individuals were observed resting on undershrub leaves along forest trails.

#### Genus *Heptodonta* Hope, 1838

The genus *Heptodonta* is represented in the Philippine islands by five species, including two recorded from Mindanao Island (Cabras et al. 2016a; Görn 2020).

**Figure 7.**
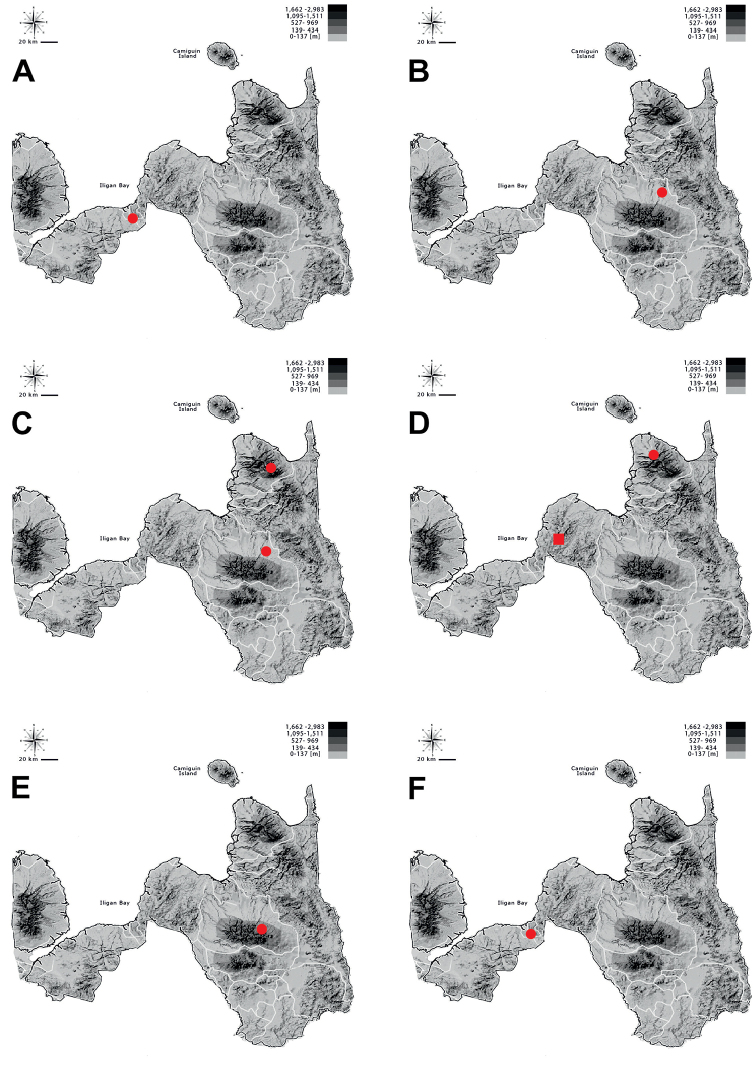
Distribution of **A**Neocollyris (Neocollyris) brevicula**B**N. (N.) emarginata**C**Neocollyris (Heterocollyris) affinis**D**N. (Heterocollyris) similior**E**N. (Heterocollyris) speciosa, and **F***Protocollyris mindanaoensis* in Northern Mindanao region.

##### Heptodonta nigrosericea

Taxon classificationAnimaliaColeopteraCicindelidae

(W. Horn, 1930)

45E8DE14-688C-59DC-956E-C2F3133052F0

Figure 8E

###### General distribution.

Species endemic to the Philippines, where it has been noted on Mindanao and Panay islands only; in Mindanao it was noted in Davao, North Mindanao, and Soccsksargen regions (Cabras et al. 2016a; Cabras and Wiesner 2016; Görn 2020; Medina et al. 2020c; Medina 2020; Pepito et al. 2020).

###### Literature data for Northern Mindanao.

**Bukidnon province**: Impasug-ong (Cabras et al. 2016a: noted as *Heptodonta lumawigi* Wiesner, 1980); Mt. Kintanglad (Görn 2020); Kabanglasan [= Cabanglasan] (Görn 2020); Intavas (Görn 2020); Silipon (Görn 2020); **Misamis Occidental province**: Mt. Malindang Range (Cabras et al. 2016a: noted as *H. lumawigi* Wiesner, 1980); **Misamis Oriental province**: Mt. Balatucan-Lumot (Cabras et al. 2016a: noted as *H. lumawigi* Wiesner, 1980); Gingoog (Görn 2020).

###### Material examined.

**Bukidnon province**: Mt. Kintanglad, 10.2014, 5♂♂ 5♀♀, leg. N. Mohagan (JWC); [no detailed locality], 1977, 1♀, leg. R. Lumawig (JWC); **Misamis Oriental province**: Gingoog, 8.5N, 125.0E, 04.1984, 7♂♂ 13♀♀ (JWC).

###### Habitat.

Species noted in shaded areas on river banks.

###### Remarks.

*Heptodonta lumawigi* is a junior synonym of this species (Görn 2020).

#### Subtribe Cicindelina Latreille, 1802

**Genus *Calomera* Motschulsky, 1862** The genus *Calomera* is represented in the Philippine islands by five species, including four recorded from Mindanao Island (Cabras et al. 2016a). Three of them have been noted from the Northern Mindanao region.

##### Calomera angulata angulata

Taxon classificationAnimaliaColeopteraCicindelidae

(Fabricius, 1798)

89FF39D0-7420-509D-9EE5-938D9E058727

Figures 3C–E , 8F

###### General distribution.

India, Nepal, Sri Lanka, Thailand, Pakistan, Afghanistan, Cambodia, Vietnam, Laos, Taiwan, Malaysia, Indonesia, China; in the Philippines the species was recorded only from Luzon (Cabras et al. 2016a) and Mindanao (new record).

###### Material examined.

**Lanao del Norte province**: Iligan City – Barangay Merila, 8°12'17"N, 124°15'24"E, 18 m a.s.l., 05–07.2017, 15♂♂ 8♀♀, 15.12.2018, 8♂♂ 3♀♀, leg. D. A. P. Acal (DAC); Iligan City – Barangay Bonbonon, 8.265458N, 124.310138E, 47 m a.s.l., 05–07.2017, 3♂♂ 1♀, leg. D. A. P. Acal (DAC); Municipality of Bacolod, Barangay Esperanza, 8°10'12"N, 124°0'22"E, 27 m a.s.l., 05–07.2017, 1♀, leg. D. A. P. Acal (DAC); Iligan City – Barangay Puga-an, sandy bank of Puga-an River, 8°13'21.3"N, 124°15'52.0"E, 29.10.2018, 3♂♂ 6♀♀, leg. C. Torres (RJC); Iligan City – Barangay Puga-an, sandy bank of Puga-an River, 8°13'29.6"N, 124°15'57.8"E, 29.10.2018, 3♂♂ 6♀♀, leg. C. Torres (RJC); Iligan City – Barangay Puga-an, rocky bank of Puga-an River, 8°13'31.5"N, 124°16'08.6"E, 29.10.2018, 2♂♂, leg. C. Torres (RJC); 29.08.2018, 1♂ 1♀, leg. D. A. P Acal (DAC); Iligan City – Barangay Tipanoy, Tubod River, 8°11'38.12"N, 124°15'25.38"E, 20 m a.s.l., 29.08.2018, 1♂, leg. D. A. P. Acal (RJC); Iligan City – Barangay Baraas, Tubod River, 8°12'40.23"N, 124°14'53.25"E, 12 m a.s.l., 17.07.2018, 4♂♂ 1♀, leg. D. A. P. Acal (RJC); Barangay Merila, Iligan City, 15.12.2018, 8♂♂ 3♀♀, leg. D. A. P. Acal (RJC).

###### Habitat.

The species occurs on sandy river banks exposed to direct sunlight (pers. obs.).

###### Remarks.

First records both from Northern Mindanao region and entire Mindanao Island. This species was observed to co-occur with *Calomera mindanaoensis*, *C. lacrymosa*, *Cylindera discreta elaphroides*, and *C. minuta*.

##### Calomera cabigasi

Taxon classificationAnimaliaColeopteraCicindelidae

Cassola, 2011

112CC1BE-461F-568A-882B-869BB62CA0F0

Figure 9A

###### General distribution.

Species endemic to Philippines where it was found only in Mindanao (Northern Mindanao region) (Cassola 2011; Cabras et al. 2016a).

###### Literature data for Northern Mindanao.

**Bukidnon province**: Impasug-ong (Cassola 2011); **Misamis Oriental province**: Gingoog City (Cassola 2011).

###### Habitat.

Species noted from river banks.

##### Calomera lacrymosa

Taxon classificationAnimaliaColeopteraCicindelidae

(Dejean, 1825)

4966DB53-5E54-51CE-815A-C6FB3900FF67

Figures 3C, E , 5D , 9B

###### General distribution.

Species endemic to Philippines where it was found in greater part of the country, including Balabac, Bucas Grande, Homonhon, Luzon, Palawan, Mindanao, Mindoro, and Sibuyan; in Mindanao Island it was noted from Davao and Northern Mindanao regions (Cabras et al. 2016a, b).

###### Literature data for Northern Mindanao.

**Lanao del Norte province**: Iligan City, Barangay Tipanoy (Cassola 2000; Cabras et al. 2016a); **Misamis Oriental province**: Municipality of Tagoloan, Tagoloan River (Cassola 2000; Cabras et al. 2016a).

###### Material examined.

**Lanao del Norte province**: Iligan City – Barangay Bonbonon, 8.265458N, 124.310138E, 47 m a.s.l., 05–07.2017, 40 exx., leg. D. A. P. Acal (DAC); Municipality of Bacolod, Barangay Esperanza, 8°10'12"N, 124°0'22"E, 27 m a.s.l., 05–07.2017, 210 exx., leg. D. A. P. Acal (DAC), 13.12.2018, 4♂♂, leg. R. Jaskuła (RJC); Iligan City – Barangay Puga-an, sandy bank of Puga-an River, 8°13'21.3"N, 124°15'52.0"E, 10.29.2018, 4♂♂ 5♀♀, leg. C. Torres (RJC); Iligan City – Barangay Puga-an, sandy bank of Puga-an River, 8°13'29.6"N, 124°15'57.8"E, 10.29.2018, 3♂♂, leg. C. Torres (RJC); Iligan City – Barangay Puga-an, rocky bank of Puga-an River, 8°13'31.5"N, 124°16'08.6"E, 10.29.2018, 7♂♂ 1♀, leg. C. Torres (RJC); Iligan City – Barangay Puga-an, 8°13'30.73"N, 124°16'6.18"E, 21 m a.s.l., 3♂♂, leg. D. A. P. Acal (RJC); Iligan City – Barangay Tipanoy, Tubod River, 8°11'38.12"N, 124°15'25.38"E, 20 m a.s.l., 29.08.2018, 1♀, leg. D. A. P. Acal (RJC); Iligan City – Barangay Merila, 8°12'17"N, 124°15'24"E, 13.12.2018, 18 m a.s.l., 4♂♂ 4♀. leg. D. A. P. Acal (RJC); Iligan City – Barangay Bonbonon, 8°16'11.69"N, 124°17'16.11"E, 11 m a.s.l., 04.11.2018, 40 exx., leg. D. A. P. Acal (DAC); Barangay Merila, Iligan City, 15.12.2018, 4♂♂ 4♀♀, leg. D. A. P. Acal (RJC); **Misamis Oriental province**: Cagayan de Oro City, Malasag Cugman, Mapawa Nature Park, 8°26'5.93"N, 124°42'12.40"E, 334 m a.s.l., 20.08.2017, 7♂♂ 1♀, leg. O. Bagona (RJC); **Misamis Occidental province**: Municipality of Lopez Jaena, 8°33'00"N, 123°46'00"E, 11.2018, 11♂♂ 4♀♀, leg. A. B. Lapore (RJC).

**Figure 8.**
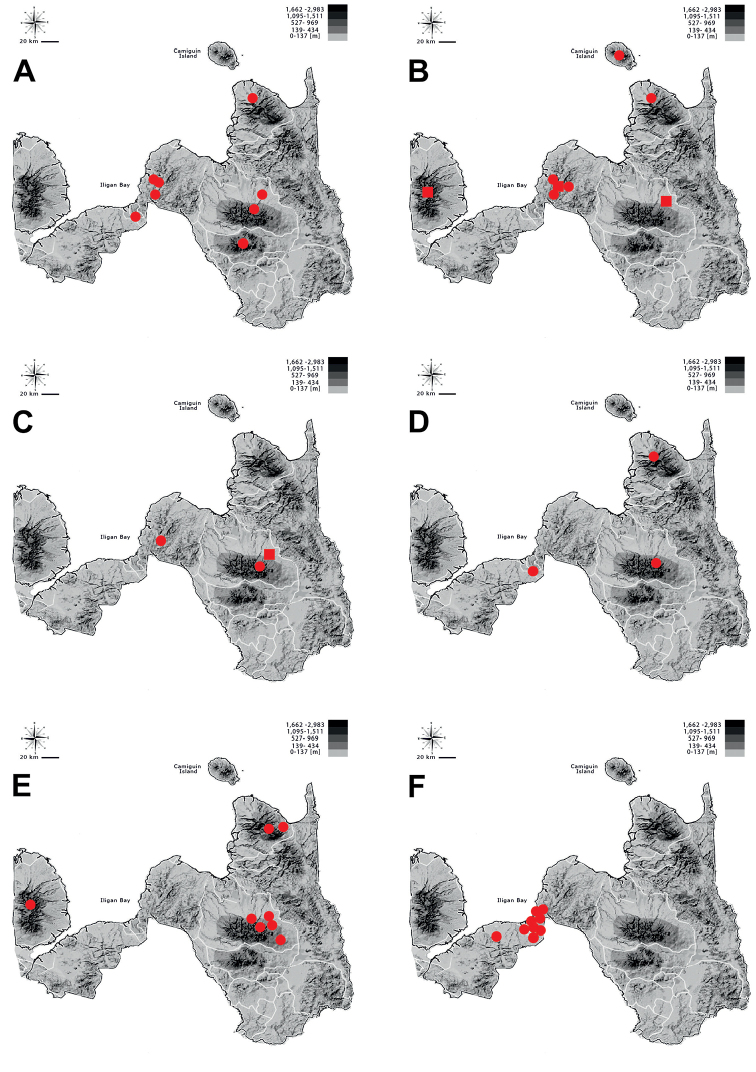
Distribution of **A***Therates coracinus coracinus***B***Therates fasciatus fasciatus* (circle) and *T. fasciatus pseudolatreillei* (square) **C***Therates fulvipennis bidentatus* (cirle) and *T. fulvipennis everetti* (square) **D**Prothyma (Symplecthyma) heteromallicollis heteromallicollis**E***Heptodonta nigrosericea*, and **F***Calomera angulata angulata* in Northern Mindanao region.

**Figure 9.**
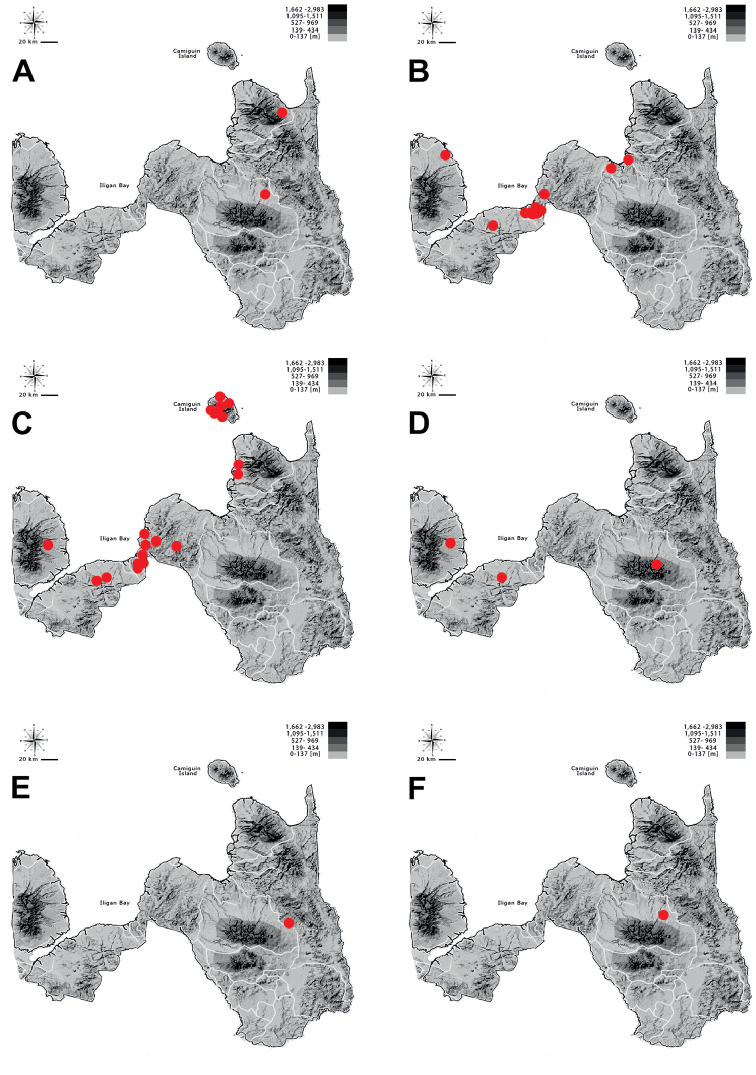
Distribution of **A***Calomera cabigasi***B***C. lacrymosa***C***C. mindanaoensis***D**Lophyra (Spilodia) striolata tenuiscripta**E**Thopeutica (Thopeutica) angulihumerosa, and **F**T. (T.) darlingtonia in Northern Mindanao region.

###### Habitat.

The species occurs on sandy river banks (pers. obs.).

###### Remarks.

At least in some areas *Calomera lacrymosa* seems to occur sympatrically or even syntopically with *C. mindanaoensis* (pers. obs.). *C. lacrymosa* was recently noted as a host of *Hexathrombium* parasitic mites (Acari: Microtrombidiidae) (Acal et al. in press).

##### Calomera mindanaoensis

Taxon classificationAnimaliaColeopteraCicindelidae

(Cassola, 2000)

E468424D-B183-51B0-BE01-35AB11CDDD5D

Figures 2A–G , 3C–F, H , 4C , 5C , 9C

###### General distribution.

Species endemic to Philippines where it was found in Mindanao (Cassola 2000; Cabras et al. 2016a, b; Cabras and Wiesner 2016) and Camiguin islands (new record); till now in Mindanao it was recorded in Davao, Northern Mindanao, Soccsksargen, and Zamboanga Peninsula regions (Cassola 2000; Cabras et al. 2016a, b; Cabras and Wiesner 2016; Pepito et al. 2020).

###### Literature data for Northern Mindanao.

**Bukidnon province**: S. Vicente, 20 km S Cagayan de Oro (Cassola 2000); Impasug-ong (Cabras et al. 2016a); **Lanao del Norte province**: Iligan City, Tipanoy (Cassola 2000); **Misamis Oriental province**: Tagoloan River, Tagoloan (Cassola 2000).

###### Material examined.

**Camiguin province**: Camiguin Island: Municipality of Mambajao, Barangay Bulok-bulok, 9°15'9"N, 124°42'31"E, 7 m a.s.l., 05–07.2017, 27♂♂ 22♀♀, leg. D. A. P. Acal (DAC); Municipality of Mambajao, Barangay Poblacion, 9°13'24"N, 124°41'47"E, 229 m a.s.l., 17.06.2017, 6♂♂ 1♀, leg. D. A. P. Acal (DAC); Municipality of Sagay, Sagay River, 28 m a.s.l., 08.07.2017, 23♂♂ 8♀♀, leg. D. A. P. Acal (DAC); Municipality of Catarman, Barangay Mainit, Looc River, 9°10'30"N, 124°40'44"E, 278 m a.s.l., 05–07.2017, 60♂♂ 31♀♀, leg. D. A. P. Acal (DAC); Municipality of Catarman, Barangay Bura, 9°10'4.7"N, 124°39'23"E, 143 m a.s.l., 17.06.2017, 3♂♂ 4♀♀, leg. D. A. P. Acal (DAC); Tuasan Falls, Looc River, Barangay Bonbon, Municipality of Catarman, 9.176009N, 124.679768E, 11.2019, 12 exx., leg. D. A. P. Acal, R. Jaskuła (RJC); **Lanao del Norte province**: Barangay Mainit (between Iligan City and Cagayan de Oro), 17–20.08.1978, 1♂ 1♀, leg. Cabides et Lobin (JWC); Municipality of Bacolod, Barangay Mati, 8°9'4"N, 124°0'57"E, 53 m a.s.l., 05–07.2017, 27♂♂ 17♀♀, leg. D. A. P. Acal (DAC); Municipality of Bacolod Barangay Esperanza, 8°10'12"N, 124°0'22"E, 27 m a.s.l., 05–08.2017, 67♂♂ 24♀♀, leg. D. A. P. Acal (DAC), 13.12.2018, 3♂♂ 1♀, leg. R. Jaskuła (RJC); Iligan City – Barangay Bonbonon, 8.265458N, 124.310138E, 47 m a.s.l., 05–08.2017, 50♂♂ 23♀♀, leg. D. A. P. Acal (DAC); Iligan City – Barangay Merila, 8°12'17"N, 124°15'24"E, 18 m a.s.l., 20.06.2017, 26♂♂ 4♀♀, leg. D. A. P. Acal (DAC); Iligan City – Barangay Puga-an, sandy bank of Puga-an River, 8°13'21.3"N, 124°15'52.0"E, 10.29.2018, 1♂ 1♀, leg. C. Torres (RJC); Iligan City – Barangay Tipanoy, Tubod River, 8°11'38.12"N, 124°15'25.38"E, 20 m a.s.l., 28.09.2018, 1♂, leg. D. A. P. Acal (DAC); Iligan City – Barangay Rogongon, Sitio Lawlawon, 8°14'51.13N, 124°25'25.31"E, 359 m a.s.l., 10.03.2019, 8 exx., leg. D. A. P. Acal (DAC); **Misamis Occidental province**: Municipality of Sinacaban, Barangay San Lorenzo, 8°17'10"N, 123°41'43"E, 800 m a.s.l., 06.09.2017, 5♂♂ 4♀♀, leg. D. A. P. Acal (DAC); **Misamis Oriental province**: Municipality of Balingasag, 8°46'9"N, 124°48'2"E, 86 m a.s.l., 07.2017, 4♂♂ 2♀♀, leg. D. A. P. Acal (DAC); Municipality of Lagonglong, 8°48'11"N, 124°48'53"E, 90 m a.s.l., 05–08.2017, 59♂♂ 29♀♀, leg. D. A. P. Acal (DAC); Municipality of Lugait, Barangay Upper Talacogon, river bank, 8°20'47.04"N, 124°16'58.80"E, 11.07.2018, 2♂♂, leg. V. M. Mirabueno (RJC).

###### Habitat.

The species was recorded as the most opportunistic according to habitat type among all Cicindelidae presented in this paper, found on sandy river banks, forest paths, coastal area, and unused compost pit near the river (pers. obs.).

###### Remarks.

At least in some areas co-occur with *C. lacrymosa*. *C. mindanaoensis* was recently noted as a host of *Hexathrombium* (Acari: Microtrombidiidae) parasitic mites (Acal et al. – in press). This species was also observed resting on undershrub plants along the trails during rainy season.

#### Genus *Lophyra* Motschulsky, 1859

The genus *Lophyra* is represented in the Philippine islands by one species, known to occur also in Mindanao Island (Cabras et al. 2016a).

#### Subgenus Spilodia Rivalier, 1961

##### Lophyra (Spilodia) striolatatenuiscripta

Taxon classificationAnimaliaColeopteraCicindelidae

(Fleutiaux, 1893)

A06E2751-358D-5318-9291-DCA070C2AB0E

Figures 3G , 5E , 9D

###### General distribution.

Subspecies known only from Indonesia and Philippines, in the second country noted only in Palawan (Cabras et al. 2016a) and Mindanao (new record).

###### Material examined.

**Bukidnon province**: Mt. Kitanglad, 11–12.2014, 1♂, leg. N. Mohagan (JWC); **Lanao del Norte province**: Municipality of Bacolod, Barangay Mati, 8°9'4"N, 124°0'57"E, 53 m a.s.l., 06.2017, 26 exx., leg. D. A. P. Acal (DAC); **Misamis Occidental province**: Municipality of Sinacaban, Barangay San Isidro, 8°17'5"N, 123°47'5"E, 269 m a.s.l., 05–07.2017, 78 exx., leg. D. A. P. Acal (DAC).

###### Habitat.

Collected along the trails of coconut field (Municipality of Bacolod, Lanao del Norte) and from cultivated corn and ginger field (Municipality of Sinacaban, Misamis Occidental) (Acal – pers. obs.).

###### Remarks.

*Lophyra striolata* is a polytypic species with wide distribution in nearly the whole Oriental region (Cassola, 2000). Four subspecies currently are known from the Philippines (ssp. striolata (Illiger, 1800), spp. *dorsolineolata* (Chevrolat, 1845), spp. *tenuiscripta* (Fleutiaux, 1893), and spp. *uniens* (Horn, 1896)) (Cabras et al. 2016a) but at least in some cases their distribution and taxonomical status should be clarified as few subspecies were noted from the same areas.

#### Genus *Thopeutica* Chaudoir, 1861

The genus *Thopeutica* is the largest tiger beetle genus in the Philippines with 31 species classified in two subgenera described to date, including 27 species in subgenus Thopeutica s. str. and four species in subgenus Philippiniella (Cabras et al. 2016a, Medina et al. 2019, 2020c). *Thopeutica* is geographically restricted to Sulawesi and the Philippines and is one of the most exclusive genera since all except two species know from the country seem to be restricted to only one island or to very few islands (Cassola and Zettel 2006).

##### Thopeutica (Thopeutica) angulihumerosa

Taxon classificationAnimaliaColeopteraCicindelidae

(Horn, 1929)

E822D53F-CDA2-545D-8BCB-D6D48EF1FADC

Figure 9E

###### General distribution.

Species endemic to the Philippines, where it was recorded from Leyte, Mindanao, and Samar; according to Cabras et al. (2016a) only general distributional data from Mindanao for this species were known, this is the first record from Northern Mindanao region.

###### Material examined.

**Bukidnon province**: Barangay Kalasungay, 8°11'28"N, 125°5'54"E, 770 m a.s.l., 14.06.2017, 1♀, leg. D.A.P. Acal (DAC).

###### Habitat.

The only specimen from Northern Mindanao studied was collected in a shaded riverine area.

##### Thopeutica (Thopeutica) darlingtonia

Taxon classificationAnimaliaColeopteraCicindelidae

Cassola & Ward, 2004

C127DB7D-F604-5E44-8FC1-B87D473B9243

Figure 9F

###### General distribution.

Species endemic to the Philippines, where it was recorded from Luzon and Mindanao; in Mindanao recorded till now only from Northern Mindanao region (Cabras et al. 2016a).

###### Literate data from Northern Mindanao region.

**Bukidnon province**: Impasug-ong (Cabras et al. 2016a).

###### Habitat.

No detailed data on habitat specificity in Northern Mindanao region but most probably occurring along rivers in shaded areas.

##### Thopeutica (Thopeutica) milanae

Taxon classificationAnimaliaColeopteraCicindelidae

Wiesner, 1992

2B0B2B9D-D4FB-5C53-9356-A3B0B7074725

Figure 10A

###### General distribution.

Species endemic to the Philippines, where it was recorded from Leyte, Luzon (?), Mindanao, and Samar; in Mindanao noted only from Northern Mindanao region (Cabras et al. 2016a).

###### Literature data from Northern Mindanao region.

**Bukidnon province**: Impasug-ong (Cabras et al. 2016a).

###### Habitat.

No detailed data on habitat specificity in Northern Mindanao region but most probably occurring along rivers in shaded areas.

##### Thopeutica (Thopeutica) virginea

Taxon classificationAnimaliaColeopteraCicindelidae

(Schaum, 1860)

D34E895E-2A12-5F47-BB5F-E78D970FD36B

Figure 10B

###### General distribution.

Species endemic to Philippines where it was recorded only from Luzon (Cabras et al. 2016a) and Mindanao (new record) islands.

###### Material examined.

**Bukidnon province**: Mt. Kitanglad, 11 –12.2014, 4 ♂♂ 3♀♀, leg. N. Mohagan (JWC).

###### Habitat.

No detailed data on habitat specificity in Northern Mindanao region but most probably occurring along rivers in shaded areas.

#### Genus *Cylindera* Westwood, 1831

The genus *Cylindera* is represented in the Philippine islands by 22 species, including five recorded from Mindanao Island (Cabras et al. 2016a).

#### Subgenus Eugrapha Rivalier, 1950

##### Cylindera (Eugrapha) minuta

Taxon classificationAnimaliaColeopteraCicindelidae

(Olivier, 1790)

51650AA3-0511-554D-B439-56CAEFDD067E

**Figure 10.**
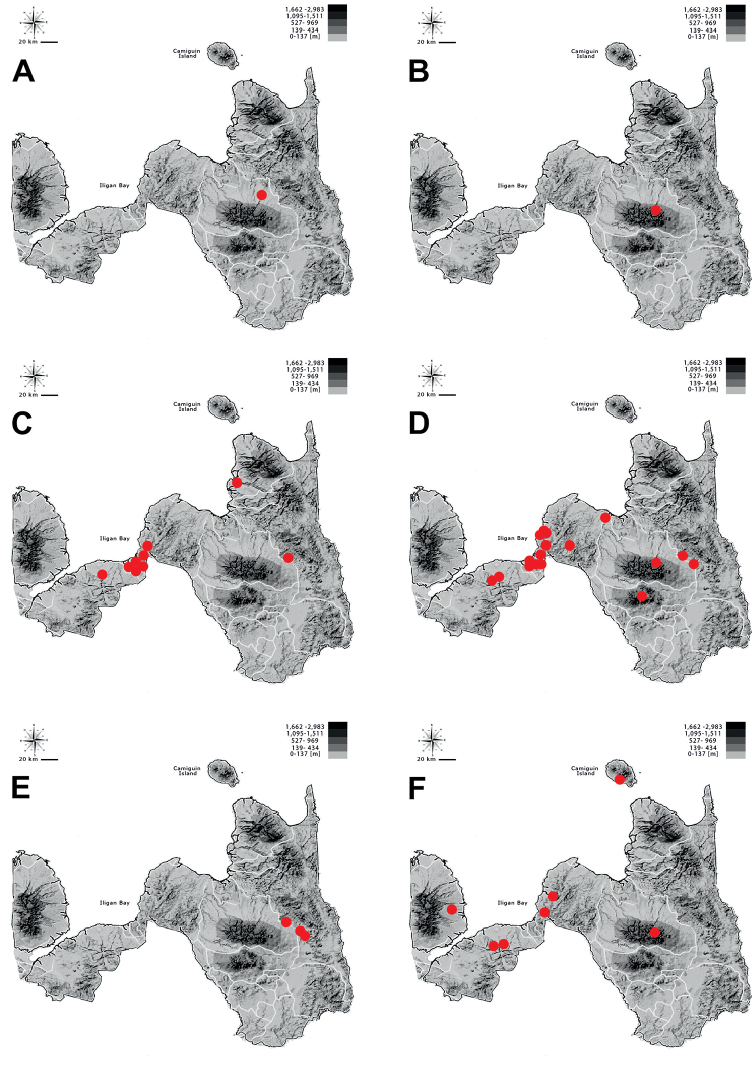
Distribution of **A**Thopeutica (T.) milanae**B**T. (Thopeutica) virginea**C**Cylindera (Eugrapha) minuta**D**C. (Ifasina) discreta elaphroides**E**C. (Ifasina) mouthiezi, and **F**C. (Ifasina) viduata in Northern Mindanao region.

Figures 2G , 3A, C–E , 5F , 10C

###### General distribution.

Species recorded till now from Bangladesh, Brunei, Cambodia, China, India, Indonesia, Laos, Malaysia, Myanmar, Nepal, Philippines, Thailand, and Vietnam (Wiesner 1992). According to Cabras et al. (2016a) in the Philippines it is known only on the basis of general distributional data; here we present the first records of this species from Mindanao.

###### Material examined.

**Bukidnon province**: Malaybalay City, Barangay Can-ayan, 8°11'31"N, 125°9'13"E, 653 m a.s.l, 15.06.2017, 1♂, leg. D.A.P. Acal (DAC); **Lanao del Norte province**: Iligan City – Barangay Bonbonon, 8.265458N, 124.310138E, 47 m a.s.l., 06.2017, 2♂♂ 2♀♀, leg. D. A. P. Acal (DAC); Iligan City – Barangay Merila, Tubod River, 8°12'17"N, 124°15'24"E, 18 m a.s.l., 05–07.2017, 108♂♂ 60♀♀, leg. D. A. P. Acal (DAC), 13.12.2018, 1♂ 1♀, leg. D. A. P. Acal (DAC); Iligan City – Barangay Puga-an, rocky bank of Puga-an River, 8°13'31.5"N, 124°16'08.6"E, 10.29.2018, 3♂♂ 2♀♀, leg. C. Torres (RJC); Iligan City – Barangay Baraas, Tubod River, 8°12'40.23"N, 124°14'53.25"E, 12 m a.s.l., 17.07.2018, 11♂♂ 6♀♀, leg. D. A. P. Acal (RJC); Iligan City – Barangay Tubod, Tubod River, 8°13'12.12"N, 124°14'56.00"E, 9 m a.s.l., 30 exx., leg. D. A. P. Acal (DAC); Municipality of Bacolod, Barangay Esperanza, 8°10'12"N, 124°0'22"E, 27 m a.s.l., 06.2017, 1♂, leg. D. A. P. Acal (DAC); Iligan City – Barangay Puga-an, 8°13'30.73"N, 124°16'6.18"E, 21 m a.s.l., 18.10.2018, 2♂♂, leg D. A. P. Acal (RJC); **Misamis Oriental province**: Municipality of Balingasag, Cabulaway River, 8°46'9"N, 124°48'2"E, 86 m a.s.l, 06–08.2017, 44♂♂ 34♀♀, leg. D. A. P. Acal (DAC).

###### Habitat.

Species occurs on sandy river banks (pers. obs.).

###### Remarks.

This species was observed to co-occur with *Calomera mindanaoensis*, *C. lacrymosa*, *C. angulata*, and/or *Cylindera discreta elaphroides* (pers. obs.).

#### Subgenus Ifasina Jeannel, 1946

##### Cylindera (Ifasina) discretaelaphroides

Taxon classificationAnimaliaColeopteraCicindelidae

(Doktouroff, 1882)

F9DB5118-A4A9-5194-9CE9-F26F29BF8C6D

Figures 2H , 3A, C–E , 5G–H , 10D

###### General distribution.

Subspecies endemic to the Philippines, recorded till now from Leyte, Mindanao, Palawan, Samar (Cabras et al. 2016a), and Cebu (Cabrera et al. 2019); in Mindanao noted from Davao (Cabras et al. 2016a, b; Cabras and Wiesner 2016) and Northern Mindanao regions (new records).

###### Material examined.

**Bukidnon province**: Mt. Kitanglad, 11–12.2014, 1♀, leg. N. Mohagan (JWC); Malaybalay City, Barangay Kalasungay, 8°11'28"N, 125°5'54"E, 770 m a.s.l., 06–08.2017, 47 exx., leg. D. A. P. Acal (DAC); Malaybalay City, Barangay Can-ayan, 8°11'31"N, 125°9'13"E, 653 m a.s.l., 06–08.2017, 226 exx., leg. D.A.P. Acal (DAC); Mt. Kalatungan, Sitio Bato, Municipality of Maramag, 11.2019., 2 exx., leg. R. Jaskuła, D. A. P. Acal (RJC); **Lanao del Norte province**: Iligan City – Barangay Bonbonon, 8.265458N, 124.310138E, 47 m a.s.l., 06.2017, 5 exx, leg. D. A. P. Acal (DAC); Iligan City – Barangay Merila, Tubod River, 8°12'17"N, 124°15'24"E, 18 m a.s.l., 05.2017, 3 exx., leg. D. A. P. Acal (DAC); Iligan City – Barangay Baraas, Tubod River, 8°12'40.23"N, 124°14'53.25"E, 12 m a.s.l., 17.07.2018, 2♀♀, leg. D. A. P. Acal (RJC); Iligan City – Barangay Tubod, Tubod River, 8°13'12.12"N, 124°14'56.00"E, 9 m a.s.l., 17.07.2018, 1 ex., leg. D.A.P. Acal (DAC); Iligan City – Barangay Puga-an, sandy bank of Puga-an River, 8°13'21.3"N, 124°15'52.0"E, 10.29.2018, 9♂♂ 2♀♀, leg. C. Torres (RJC); Iligan City – Barangay Puga-an, sandy bank of Puga-an River, 8°13'29.6"N, 124°15'57.8"E, 10.29.2018, 3♂♂, leg. C. Torres (RJC); Iligan City – Barangay Tipanoy, Tubod River, 8°11'38.12"N, 124°15'25.38"E, 20 m a.s.l., 29.08.2018, 4♂♂ 1♀, leg. D. A. P. Acal (RJC); Municipality of Bacolod, Barangay Esperanza, 8°10'12"N, 124°0'22"E, 27 m a.s.l., 05.2017, 1 ex., leg. D. A. P. Acal (DAC), 13.12.2018, bank of river, 2♂♂, leg. R. Jaskuła (RJC); Municipality of Bacolod, Barangay Mati, 8°9'4"N, 124°0'57"E, 53 m a.s.l., 13.12.2018, 2♂♂, leg. R. Jaskuła (RJC); Iligan City – Barangay Rogongon, Sitio Lawlawon, 8°14'51.13N, 124°25'25.31"E, 359 m a.s.l., 10.03.2019, 10 exx., leg. D. A. P. Acal (DAC); **Misamis Oriental province**: Cagayan de Oro City, Malasag Cugman, Mapawa Nature Park, 8°26'5.93"N, 124°42'12.40"E, 334 m a.s.l., 20.08.2017, 9♂♂ 7♀♀, leg. O. Bagona (RJC); Municipality of Lugait, Barangay Upper Talacogon, river bank, 8°20'47.04"N, 124°16'58.80"E, 08.11.2018, 2♂♂ 2♀♀, leg. V. M. Mirabueno (RJC), 01.12.2018, 4♂♂ 1♀, leg. V. M. Mirabueno (RJC); Municipality of Lugait, Barangay Lower Talacogon, river bank, 8°20'47.04"N, 124°16'58.80"E, 03.12.2018, 2♂♂ 1♀, leg. V. M. Mirabueno (RJC); Municipality of Lugait, Barangay Aya-Aya, river bank, 8°20'00.16"N, 124°18'36.77"E, 07.11.2018, 2♂♂, leg. V. M. Mirabueno (RJC).

###### Habitat.

A riverine tiger beetles species recorded on sandy bank (per. obs.).

###### Remarks.

Although *C. discreta elaphroides* is active mainly during sunlight hours on the river banks, it was also noted as species actively hunting during heavy rain on vertical surfaces (Cabrera et al. 2019). Some specimens were also observed resting on the leaves of *Pennisetum* sp. along the river (pers. obs.).

##### Cylindera (Ifasina) mouthiezi

Taxon classificationAnimaliaColeopteraCicindelidae

Dheurle, 2015

9916B848-89BB-54F4-BA9D-979FF3BF6101

Figures 2H , 10E

###### General distribution.

Species endemic to Philippines (Cabras et al. 2016) where it has been recorded only from Mindanao, where it was found only from Davao and Northern Mindanao regions till now (Dheurle 2015, 2017; Cabras et al. 2016b).

###### Literature data from Northern Mindanao region.

**Bukidnon province**: Cabanglasan (Dheurle 2015, 2017); Panamokan (Dheurle 2017).

###### Material examined.

**Bukidnon province**: Cabanglasan, 06.2014, 1♂ (JWC); Panamokan, 06.2014, 1♀ (JWC); Barangay Kalasungay, 8°11'28"N, 125°5'54"E, 770 m a.s.l., 14.06.2017, 8 exx., leg. D. A. P. Acal (DAC, RJC).

###### Habitat.

All specimens known for us from Northern Mindanao region were collected in a shaded riverine area.

###### Remarks.

This species co-occurs with *Cylindera discreta elaphroides* and *Thopeutica angulihumerosa*.

##### Cylindera (Ifasina) viduata

Taxon classificationAnimaliaColeopteraCicindelidae

(Fabricius, 1801)

7006C54E-0014-59A8-B8AE-E75BF63B30E2

Figures 3G , 10F

###### General distribution.

Species recorded from Bangladesh, Cambodia, China, India, Indonesia, Laos, Malaysia, Myanmar, Nepal, Papua New Guinea, Philippines, Thailand, Vietnam; in the Philippines is was noted from the following islands: Leyte, Mindanao, Palawan, and Tawi-tawi; in Mindanao recorded only on the basis of general information (Cabras et al. 2016a); here we provide the first records from Northern Mindanao region.

###### Material examined.

**Bukidnon province**: Mt. Kitanglad, 11–12.2014, 1♀, leg. N. Mohagan (JWC); **Camiguin province**: Camiguin Island, Municipality of Sagay, Barangay Bonbon, Sagay River, 28 m a.s.l., 08.07.2017, 2♂♂, leg. D. A. P. Acal (DAC); **Lanao del Norte province**: Iligan City, Barangay Bonbonon, 8.265458N, 124.310138E, 47 m a.s.l., 29.05.2017, 3♂♂ 2♀♀, leg. D. A. P. Acal (DAC); 11.2019, 1ex., leg. R. Jaskuła (RJC); Iligan City – Barangay Esperanza, 8°10'12"N, 124°0'22"E, 27 m a.s.l., 08.06.2017, 3♀♀, leg. D. A. P. Acal (DAC); Municipality of Bacolod, Barangay Mati, 8°9'4"N, 124°0'57"E, 53 m a.s.l., 05–07.2017, 22♂♂ 13♀♀, leg. D. A. P. Acal (DAC), 13.12.2018, 2♂♂, leg. D. A. P. Acal (DAC); **Misamis Occidental province**: Municipality of Sinacaban, Barangay San Isidro, 8°17'5"N, 123°47'5"E, 269 m a.s.l., 05–07.2017, 8♂♂ 5♀♀, leg. D. A. P. Acal (DAC); **Misamis Oriental province**: Mambuntan Falls, Barangay Lubilan, Naawan Municipality, 8.412300N, 124.351642E, 11.2019, 2exx., ad lucem, leg. R. Jaskuła (RJC).

###### Habitat.

Species noted from trails along riverine areas, river banks, cultivated corn and ginger fields (pers. obs.).

###### Remarks.

This species co-occurs with *Lophyra striolata* in agricultural fields (pers. obs.).

### Provisional key to tiger beetle species known to occur in the Northern Mindanao Region

**Table:** 

1	Metepisternum narrow, with grooves anteriorly; mesepisternum strongly elongated	**2**
–	Metepisternum relatively broad, without anterior grooves; mesepisternum usually short	**13**
2	Outer margin of mandible without tooth; labrum 6-dentate; humeral angles of elytra and hind wings absent	**3 Tribe Collyridini Brullé, 1834, Subtribe Tricondylina Naviaux, 1991**
–	Outer margin of mandible with tooth; labrum 7-dentate; humeral angles and hind wings present	**7 Tribe Collyridini Brullé, 1834, Subtribe Collyridina Brullé, 1834**
3	Base of interocular cavity at same level as neck; no distinct transverse line between neck and occiput; pronotum never both long and subrectangular; body length from 13 to 28 mm	**4 Genus Tricondyla Latreille, 1822, Subgenus Tricondyla s. str.**
–	Base of interocular cavity slightly higher than neck or not exactly on same extension; presence of a distinct transverse mark between neck and occiput; temples abruptly shaped from dorsal view; body length less than 17 mm, habitus very slender	**6 Genus Tricondyla Latreille, 1822, Subgenus Stenotricondyla Naviaux, 2002**
4	Very robust species with body length 19–24 mm; elytral sculpture may be almost smooth or granular	**Tricondyla (Tricondyla) aptera punctipennis Chevrolat, 1841**
–	Smaller and narrower species with body length 15–20 mm; sculpture covering entire elytra (but decreasing at apex) or posterior half part almost smooth	**5**
5	Elytral sculpture deeper and covering the entire surface but decreasing at apex; median lobe of aedeagus with tip particularly acute	**Tricondyla (Tricondyla) gracilis Naviaux, 2002**
–	Elytral sculpture not regularly distributed with the posterior half almost smooth; median lobe of aedeagus with tip less acute	**Tricondyla (Tricondyla) elongata Horn, 1906**
6	Generally smaller species (usually between 13 and 15.5 mm), aedeagus not longer than 2.5 mm	**Tricondyla (Stenotricondyla) cyanipes Eschscholtz, 1829**
–	Larger species, usually with body length between 14 and 17 mm; aedeagus at least 3 mm long	**Tricondyla (Stenotricondyla) cavifrons Schaum, 1862**
7	Labrum very short; body smaller than 9 mm, slender; sculpture of elytra shallow, dense and uniform	**Genus *Protocollyris* Mandl, 1975, *Protocollyris mindanaoensis* (Mandl, 1974)**
–	Labrum longer	**8 Genus *Neocollyris* Horn, 1901**
8	Generally smaller and slender species (between 9.5 and 13.5 mm)	**9 Genus Neocollyris Horn, 1901, Subgenus Neocollyris s. str.**
–	Larger and robust species with body length between 17.5 and 23 mm	**11 Genus Neocollyris Horn, 1901, Subgenus Heterocollyris Naviaux, 1995**
9	Antennae short, reaching basal half of pronotum; color dark, not bright blue	**Neocollyris (Neocollyris) brevicula Naviaux, 1994**
–	Antennae longer, reaching base of pronotum; color bright blue, sometimes with violet reflections	**10**
10	Vertex dilated behind eyes; pronotum short; tip of aedeagus rounded	**Neocollyris (Neocollyris) emarginata (Dejean, 1825)**
–	Vertex not dilated behind eyes, pronotum longer; tip of aedeagus acute	**Neocollyris (Neocollyris) albitarsis (Erichson, 1834)**
11	Pronotum strongly constricted in front; aedeagus sigmoid in lateral view	**Neocollyris (Heterocollyris) speciosa (Schaum, 1863)**
–	Pronotum lesser constricted in front; aedeagus not sigmoid in lateral view	**12**
12	Elytral sculpture dense and fine, less creased near suture	**Neocollyris (Heterocollyris) similior (Horn, 1893)**
–	Elytral sculpture not very coarse; creased along sutural margin	**Neocollyris (Heterocollyris) affinis (Horn, 1892)**
13	Galea of maxilla reduced, one segmented; 4^th^ tarsal segment very shortened, with 5^th^ segment inserted toward the middle of its upper side; labrum long	**14 Tribe Cicindelini Latreille, 1802, Subtribe Theratina Horn, 1910**
–	Galea of maxilla two-segmented; 4^th^ tarsal segment rarely shortened, 5^th^ segment always inserted apically; labrum often short	**18**
14	Clypeus with two sensitive hairs	**15**
–	Clypeus without sensitive hairs	**17**
15	Elytra completely shiny black	**16**
–	Elytra black with brownish maculation	***Therates fulvipennis bidentatus* Chaudoir, 1861**
16	Metasternum yellow	***Therates fulvipennis everetti* Erichson, 1834**
–	Metasternum black	***Therates coracinus coracinus* Erichson, 1834**
17	Black maculation of elytra does not reach the furrow behind the basal hump	***Therates fasciatus fasciatus* (Fabricius, 1801)**
–	Black maculations of elytra covers the furrow behind the basal hump	***Therates fasciatus pseudolatreillei* Horn, 1928**
18	Head, pronotum, pro- and mesosternum, base of abdomen and elytra glabrous	**19 Tribe Cicindelini Latreille, 1802, Subtribe Prothymina Horn, 1906**
–	Either head, pronotum, pro- and mesosternum, base of sternum or base of elytra setose	**20 Tribe Cicindelini Latreille, 1802, Subtribe Cicindelina Latreille, 1802**
19	Body ventrally almost entirely glabrous except for fringe of setae on free lateral margin of hind coxae, elytra immaculate	***Heptodonta nigrosericea* Horn 1930 (= *lumawigi* Wiesner, 1980)**
–	Body ventrally entirely glabrous	**Prothyma (Symplecthyma) heteromallicollis heteromallicollis Horn, 1909**
20	Flagellum of inner sac of aedeagus coiled in a sagittal plane	**20 Genus *Cylindera* Westwood, 1831**
–	Flagellum complexly coiled on both sides of the inner sac	**24**
21	Elytra with complete humeral lunule	**Cylindera (Eugrapha) minuta (Olivier, 1790)**
–	Humeral lunule absent, himeral maculations, if present, split in two dots	**22 Genus Cylindera Westwood, 1831, Subgenus Ifasina Jeannel, 1946**
22	Elytra without any humeral maculations	**Cylindera (Ifasina) viduata (Fabricius, 1801)**
–	Humeral maculations present	**23**
23	Humeral maculations consists of a small posthumeral dot only, humeral dot absent	**Cylindera (Ifasina) discreta elaphroides (Doktouroff, 1882)**
–	Humeral maculations constist of a large humeral and a large posthumeral dot	**Cylindera (Ifasina) mouthiezi Dheurle, 2015**
24	Flagellum of inner sac of aedeagus with more than four windings	**25 Genus *Thopeutica* Schaum, 1861**
–	Flagellum with less than four windings	**28**
25	Prothorax mostly glabrous, setation restricted to pleurosternal suture or along the anterior margin	**26**
–	Pronotum with lateral margins and/or lateral angles variably setose	**27**
26	Labrum with 10–14 submarginal setae; females without elytral mirror	**Thopeutica (Thopeutica) virginea (Schaum, 1860)**
–	Labrum with 8 submarginal and mesal setae; females with elytral mirror spot	**Thopeutica (Thopeutica) milanae Wiesner, 1992**
27	Elytra with humeral callus; prosternum distinctly longer than wide	**Thopeutica (Thopeutica) angulihumerosa (Horn, 1929)**
–	Elytra without humeral callus; prosternum slightly longer than wide	**Thopeutica (Thopeutica) darlingtonia Cassola et Ward, 2004**
28	Labrum with more than ten marginal setae	**29 Genus *Calomera* Motschulsky, 1862**
–	Labrum with four to eight marginal setae	**Genus *Lophyra* Motschulsky, 1859, Lophyra (Spilodia) striolata tenuiscripta (Fleutiaux, 1893)**
29	Elytral maculation consists of humeral and apical lunule marginal band and middle band, which all are more or less connected with one another; female elytra expanded laterally	***Calomera angulata angulata* (Fabricius, 1798)**
–	Elytral maculation consists of apical lunule and five or six dots; female elytra are not expanded laterally	**30**
30	Elytra with apical lunule and five dots (humeral, subhumeral, submarginal, discal and another submarginal one below the other submarginal dot); elytra velvety black, without visible punctuation throughout	***Calomera cabigasi* Cassola, 2011**
–	Elytra with apical lunule and six dots (as mentioned above, plus an upper discal dot); elytra with visible punctuation	**31**
31	Elytra greenish or bluish, with blue green punctuation; aedeagus short, with a tiny hook shaped tip	***Calomera lacrymosa* (Dejean,1825)**
–	Elytra darker, nearly velvety black, punctuation nearly not visible in apical half of elytra; aedeagus longer, ending in a long straight apical beak	***Calomera mindanaoensis* (Cassola, 2000)**

## Discussion

### Tiger beetle fauna of Northern Mindanao vs. fauna of the entire island and country

Thirty species (including two with two subspecies each) classified in ten genera are actually known from Northern Mindanao region (Table 2, Figures 5–9), which constitute 56% of Cicindelidae fauna of Mindanao and 21% of the Philippines. Three of these species are known as endemics of Mindanao Island (*Neocollyris similior*, *Calomera cabigasi*, and Cylindera (Ifasina) mouthiezi), one more (*Calomera mindanaoesis*) is restricted only to Mindanao and Camiguin islands, while 22 taxa occur only in the Philippines (Cabras et al. 2016a). Among the recorded taxa, *Neocollyris speciosa*, *Calomera angulata*, *Cylindera minuta*, and *Lophyra striolata tenuiscripta*, *Thopeutica virginea* were noted for the first time from Mindanao island, and moreover, an additional seven species had not been reported from Northern Mindanao region before (*Tricondyla cavifrons*, *Neocollyris similior*, *Prothyma heteromallicollis heteromallicollis*, *Thopeutica angulihumerosa*, *Cylindera discreta elaphroides*, *C. mouthiezi*, *C. viduata*). The highest number of taxa was noted from Bukidnon (24 species, 80% of Northern Mindanao fauna) Misamis Oriental (15 species, 50% of fauna), and Lanao del Norte provinces (14 species, 47% of fauna). Misamis Occidental and Camiguin provinces were characterized by 23% and 7% of fauna respectively (Table 2). The number of recorded Cicindelidae taxa seems to depend on the surface area of the province, as Bukidnon is the largest one and Camiguin is the smallest area. Since not all types of habitats were studied in particular provinces, additional tiger beetle species inhabiting in Northern Mindanao region are expected to be found with more extensive fieldwork in the future.

**Table 2.** Distribution of tiger beetles in administrative provinces of Northern Mindanao region.

Species	Provinces of Northern Mindanao region				
	Bukidnon	Camiguin	Lanao del Norte	Misamis Occidental	Missamis Oriental
Tricondyla (Tricondyla) aptera punctipennis Chevrolat, 1841	+		+		
Tricondyla (Tricondyla) elongata Horn, 1906	+		+	+	+
Tricondyla (Tricondyla) gracilis Naviaux, 2002					+
Tricondyla (Stenotricondyla) cyanipes Eschscholtz, 1829					+
Tricondyla (Stenotricondyla) cavifrons Schaum, 1862	+		+		
Neocollyris (Neocollyris) albitarsis (Erichson, 1834)	+				
Neocollyris (Neocollyris) brevicula Naviaux, 1994			+		
Neocollyris (Neocollyris) emarginata (Dejean, 1825)	+				
Neocollyris (Heterocollyris) affinis (Horn, 1892)	+				+
Neocollyris (Heterocollyris) similior (Horn, 1893)			+		+
Neocollyris (Heterocollyris) speciosa (Schaum, 1863)	+				
*Protocollyris mindanaoensis* (Mandl, 1974)			+		
*Therates coracinus coracinus* Erichson, 1834	+		+		+
*Therates fasciatus fasciatus* (Fabricius, 1801)	+	+	+		+
*Therates fasciatus pseudolatreillei* Horn, 1928	+		+	+	+
*Therates fulvipennis bidentatus* Chaudoir, 1861	+		+		
*Therates fulvipennis everetti* Erichson, 1834	+				
Prothyma (Symplecthyma) heteromallicollis heteromallicollis Horn, 1909	+		+		+
*Heptodonta nigrosericea* (W. Horn, 1930)	+			+	+
*Calomera angulata angulata* (Fabricius, 1798)			+		
*Calomera cabigasi* Cassola, 2011	+				+
*Calomera lacrymosa* (Dejean,1825)			+	+	+
*Calomera mindanaoensis* (Cassola, 2000)	+	+	+	+	+
Lophyra (Spilodia) striolata tenuiscripta (Fleutiaux, 1893)	+		+	+	
Thopeutica (Thopeutica) angulihumerosa (Horn, 1929)	+				
Thopeutica (Thopeutica) darlingtonia Cassola et Ward, 2004	+				
Thopeutica (Thopeutica) milanae Wiesner, 1992	+				
Thopeutica (Thopeutica) virginea (Schaum, 1860)	+				
Cylindera (Eugrapha) minuta (Olivier, 1790)	+		+		+
Cylindera (Ifasina) discreta elaphroides (Doktouroff, 1882)	+		+		+
Cylindera (Ifasina) mouthiezi Dheurle, 2015	+				
Cylindera (Ifasina) viduata (Fabricius, 1801)	+	+	+	+	+
**Total**	**25**	**3**	**18**	**7**	**16**

### Tiger beetles and their habitats

Among Cicindelidae taxa recorded in Northern Mindanao region both epigeic (*Calomera*, *Cylindera*, *Heptodonta*, *Lophyra*, *Prothyma*, *Thopeutica*) and arboreal (*Therates*, *Neocollyris*, *Protocollyris*, *Tricondyla*) species were noted. Most of the epigeic species are recognized as riverine tiger beetles (all *Calomera* and most *Cylindera* except *C. viduata*, as well as *Heptodonta nigrosericea*, *Lophyra*, *Thopeutica*) occurring on sandy and sunny banks or on shaded banks of medium and large rivers. Among the epigeic Cicindelidae only *Cylindera viduata*, *Prothyma heteromallicollis heteromallicollis*, and some *Lophyra striolata tenuiscripta* were noted as forest beetles occupying different sandy areas. Our data from Northern Mindanao region confirm observations both from other regions of Mindanao (Cabras et al. 2016b; Cabras and Wiesner 2016; Medina 2020; Medina et al. 2020; Pepito et al. 2020) and different parts of the world including e.g., some regions of North America (Pearson et al. 1997), Africa (Jaskuła 2015; Jaskuła et al. 2015; Jaskuła and Rewicz 2015; Jaskuła and Płociennik 2020), Asia (Dangalle et al. 2014) or Europe (Jaskuła 2011; Jaskuła et al. 2019), as tiger beetles are known to prefer riverine habitats not only because of adequate water and food resources but also for protection from predators and human disturbances (Bhargav and Uniyal 2008). In many regions of the world the highest diversity and species richness of epigeic tiger beetles are noted mostly on lowland areas that had a variety of habitats such as coastal areas, river banks, grasslands, and sand dunes attractive for tiger beetles (e.g., Pearson and Cassola 1992; Pearson et al. 1997; Jaskuła 2011, 2015; Dangalle et al. 2014; Jaskuła et al. 2019; Jaskuła and Płociennik 2020). On the other hand, in the tropical regions large number of Cicindelidae are typical arboreal taxa (e.g., Wiesner 1992; Pearson and Vogler 2001; Moravec 2007; Dangalle 2018) and large forests, especially natural ones, are characterized by high species diversity of such tiger beetles. In Northern Mindanao region, where more than 60% of its entire area is classified as forest land, 48% of all recorded Cicindelidae taxa are noted as arboreal taxa (Table 2). This number is expected to increase in the future as several additional arboreal species are known from other parts of Mindanao, including areas located close to the administrative border of Northern Mindanao region (Cabras et al. 2016a). Moreover, in case of some species, only general distributional data from Mindanao are known (e.g., *Protocollyris okajimai* Mandl, 1982, *Neocollyris rugei* Horn, (1892) *N. erichsoni* (Horn, 1892), *N. chaudoiri* (Horn, 1892) (Naviaux 1994)), it cannot be excluded that some of these taxa were collected (and actually inhabit) in the Northern Mindanao region. On the other hand it is necessary to note that many areas in Mindanao, including Northern Mindanao region, are under large impacts of human activities, and as a result many tiger beetle habitats are regularly destroyed. Forest destruction, including deforestation in all sorts and forms such as illegal logging, mining, agricultural expansion, quarrying, over-extraction of plant biota for fuel and other domestic uses, or conversion of land into human settlement are among the rampant problems in the area (Magdalena 1996; Carandang et al. 2012).

## Conclusions

Present data on diversity and distribution of Cicindelidae of Northern Mindanao region clearly suggest that the area (especially riverine habitats and forests) is unique for tiger beetle fauna which includes a significant number of both species endemic to Mindanao and to the entire country. Moreover, the lack of data on Cicindelidae in many areas in Mindanao Island and in the country is evident, and for many species, only single records are known. As the region is characterized by a large mosaic of still poorly explored habitats (e.g., forests in the mountains, upper parts of river systems), and more than 12% of all species noted from Mindanao were discovered and described as new for science only during last two decades, it should be expected that future studies will provide many new and important distributional data and probably will describe new Cicindelidae taxa.

## Supplementary Material

XML Treatment for Tricondyla (Tricondyla) apterapunctipennis

XML Treatment for Tricondyla (Tricondyla) elongata

XML Treatment for Tricondyla (Tricondyla) gracilis

XML Treatment for Tricondyla (Stenotricondyla) cyanipes

XML Treatment for Tricondyla (Stenotricondyla) cavifrons

XML Treatment for Neocollyris (Neocollyris) albitarsis

XML Treatment for Neocollyris (Neocollyris) brevicula

XML Treatment for Neocollyris (Neocollyris) emarginata

XML Treatment for Neocollyris (Heterocollyris) affinis

XML Treatment for Neocollyris (Heterocollyris) similior

XML Treatment for Neocollyris (Heterocollyris) speciosa

XML Treatment for Protocollyris mindanaoensis

XML Treatment for Therates coracinus coracinus

XML Treatment for Therates fasciatus fasciatus

XML Treatment for Therates fasciatus pseudolatreillei

XML Treatment for Therates fulvipennis bidentatus

XML Treatment for Therates fulvipennis everetti

XML Treatment for Prothyma (Symplecthyma) heteromallicollisheteromallicollis

XML Treatment for Heptodonta nigrosericea

XML Treatment for Calomera angulata angulata

XML Treatment for Calomera cabigasi

XML Treatment for Calomera lacrymosa

XML Treatment for Calomera mindanaoensis

XML Treatment for Lophyra (Spilodia) striolatatenuiscripta

XML Treatment for Thopeutica (Thopeutica) angulihumerosa

XML Treatment for Thopeutica (Thopeutica) darlingtonia

XML Treatment for Thopeutica (Thopeutica) milanae

XML Treatment for Thopeutica (Thopeutica) virginea

XML Treatment for Cylindera (Eugrapha) minuta

XML Treatment for Cylindera (Ifasina) discretaelaphroides

XML Treatment for Cylindera (Ifasina) mouthiezi

XML Treatment for Cylindera (Ifasina) viduata
